# Monitoring the Formation of Nickel-Poor and Nickel-Rich Oxide Cathode Materials for Lithium-Ion Batteries with Synchrotron Radiation

**DOI:** 10.1021/acs.chemmater.2c02639

**Published:** 2023-01-31

**Authors:** Bixian Ying, Jack R. Fitzpatrick, Zhenjie Teng, Tianxiang Chen, Tsz Woon Benedict Lo, Vassilios Siozios, Claire A. Murray, Helen E. A. Brand, Sarah Day, Chiu C. Tang, Robert S. Weatherup, Michael Merz, Peter Nagel, Stefan Schuppler, Martin Winter, Karin Kleiner

**Affiliations:** † MEET, Battery Research Center, University of Muenster, Corrensstr. 46, 48149Münster, Germany; ‡ Department of Chemistry, Molecular Sciences Research Hub, Imperial College London, White City Campus, 82 Wood Lane, W12 0BZLondon, U.K.; § Department of Applied Biology and Chemical Technology, The Hong Kong Polytechnic University, Hunghom, 999077Kowloon, Hong Kong, China; ∥ Diamond Light Source Ltd, Harwell Science & Innovation Campus, Didcot, OX11 0DEOxfordshire, U.K.; ⊥ Australian Synchrotron ANSTO, 800 Blackburn Rd., Clayton, 3168Victoria, Australia; # Department of Materials, University of Oxford, Parks Road, OX1 3PHOxford, U.K.; ¶ Institute for Quantum Materials and Technologies, Karlsruhe Institute of Technology, 76021Karlsruhe, Germany; ∇ Karlsruhe Nano Micro Facility (KNMFi), Karlsruhe Institute of Technology (KIT), 76344Eggenstein-Leopoldshafen, Germany; ○ Helmholtz-Institute Münster, Forschungszentrum Jülich GmbH, 48149Muenster, Germany

## Abstract

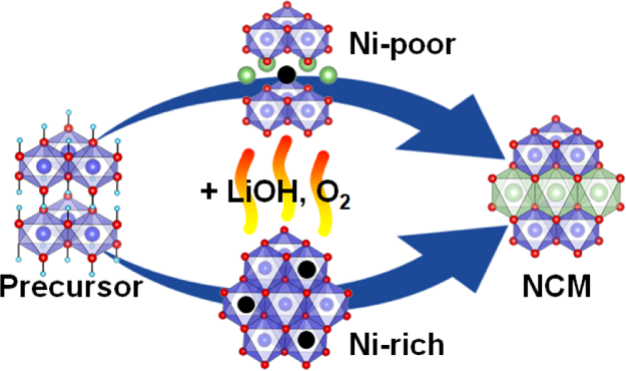

The syntheses of Ni-poor (NCM111, LiNi_1/3_Co_1/3_Mn_1/3_O_2_) and Ni-rich (NCM811 LiNi_0.8_Co_0.1_Mn_0.1_O_2_) lithium transition-metal oxides (space group *R*3̅m) from hydroxide precursors (Ni_1/3_Co_1/3_Mn_1/3_(OH)_2_, Ni_0.8_Co_0.1_Mn_0.1_(OH)_2_) are investigated using *in situ* synchrotron powder diffraction and near-edge X-ray absorption fine structure spectroscopy. The development of the layered structure of these two cathode materials proceeds *via* two utterly different reaction mechanisms. While the synthesis of NCM811 involves a rock salt-type intermediate phase, NCM111 reveals a layered structure throughout the entire synthesis. Moreover, the necessity and the impact of a preannealing step and a high-temperature holding step are discussed.

## 1 Introduction

Layered transition-metal oxides (LiMeO_2_) such as Ni-rich NCMs (LiNi_*x*_Co_*y*_Mn_1–*x*–*y*_O_2_, 0 ≤ *x*, *y* ≤ 1.0) are promising cathode materials in lithium-ion batteries due to their high specific capacities (up to 220 mA h/g). Me/Li disorder has been considered one of the main factors contributing to the rapid capacity and voltage fade, poor thermal stability, and irreversible reactions on the surface, which still limit their application in next-generation Li-ion batteries.^1−4^

Ni-rich NCMs can be synthesized from a transition-metal hydroxide precursor (Ni_*x*_Co_*y*_Mn_1–*x*–*y*_(OH)_2_) obtained from a precipitation process by calcination with LiOH at temperatures of ≈800 °C. The applied heat profile may include a holding step at 400–500 °C, and the calcination atmosphere can vary (air or pure oxygen).^5−8^ At high temperatures, the ordered and disordered rhombohedral structures of the NCMs coexist.^8,9^ Due to the similar radii of Ni^2+^ (0.69 Å) and Li^+^ (0.76 Å),^3^ Ni^2+^ present in the precursor can move into the lithium layer (Li 3b site). At the same time, Li ions occupy Me 3a sites of the rhombohedral structure, a phenomenon termed cation disorder. Despite many reports on various strategies to lower the cation disorder, such as core–shell structures,^10−12^ concentration gradient structures,^13−15^ cationic,^3,16,17^ and anionic substitution,^18−20^ execution of these strategies to obtain desired materials remains challenging due to the complexity of the structural changes occurring upon the synthesis of Ni-rich NCMs. *In situ* synchrotron X-ray powder diffraction (SXPD) is a powerful tool for tracking the structural evolution of NCMs during synthesis.^16,21,22^ Zheng *et al.*(1) and Zhang *et al.*(22) revealed that the symmetry breaking and reconstruction of the NiO_6_ octahedra plays an essential role in lowering cation disorder upon calcination. Hua *et al.*(23) elucidated the reaction inhomogeneity across particles upon the transformation from the hydroxide precursor into the final rhombohedral structure of a series of layered oxides using *in situ* X-ray diffraction (XRD), high-angle annular dark-field scanning transmission electron microscopy (TEM), high-resolution TEM, and other techniques. Bianchini *et al.*(24) monitored the calcination and decomposition of LiNiO_2_ (LNO) at high temperatures and demonstrated a complex phase transition starting from a compressed structure within a rock-salt framework. Park *et al.*(25) unraveled with an *in situ* TEM study that the synthesis of NCM622 is governed by the kinetic competition between the inner thermal decomposition and the outer lithiation of the precursor.

In addition to the processes discussed above,^1,22−25^ this work shows that the Ni oxidation from Ni^2+^ to Ni^3+^ determines the final performance of the materials.^26−28^ An incomplete oxidation leads to a poor rate and cycling stability, and the preannealing step is a key factor to ensure proper oxidation. *In situ* synchrotron and laboratory-based X-ray powder diffraction (SXPD and LXPD) coupled with mass spectrometry (MS) as well as near-edge X-ray absorption spectroscopy (NEXAFS) is used to study the evolution of the layered structures, the oxidation of the transition metals, and water loss upon calcination of Ni-poor (NCM111) and Ni-rich (NCM811) layered oxides. Applying different holding steps upon calcination, that is, using a precalcination step at 500 °C, a holding step at 800 °C, and the combination of both helps to understand crucial parameters to obtain a defect-free and highly ordered rhombohedral structure. This work provides significant insights into the mechanisms underlying Ni-rich NCM synthesis, an inspiration for the design of other Ni-rich high-energy-density cathode materials. Moreover, after long-term cycling, degradation reactions lead to a NiO phase formation on the surface.^29−31^ The reactivation of spent cathode materials requires both lithium and transition-metal recovery processes.^32,33^ Our work serves as a revelation for recycling works of cathode materials, thus preventing environmental pollution.

## 2 Experimental Section

### 2.1 Material Synthesis

**Figure 1:**
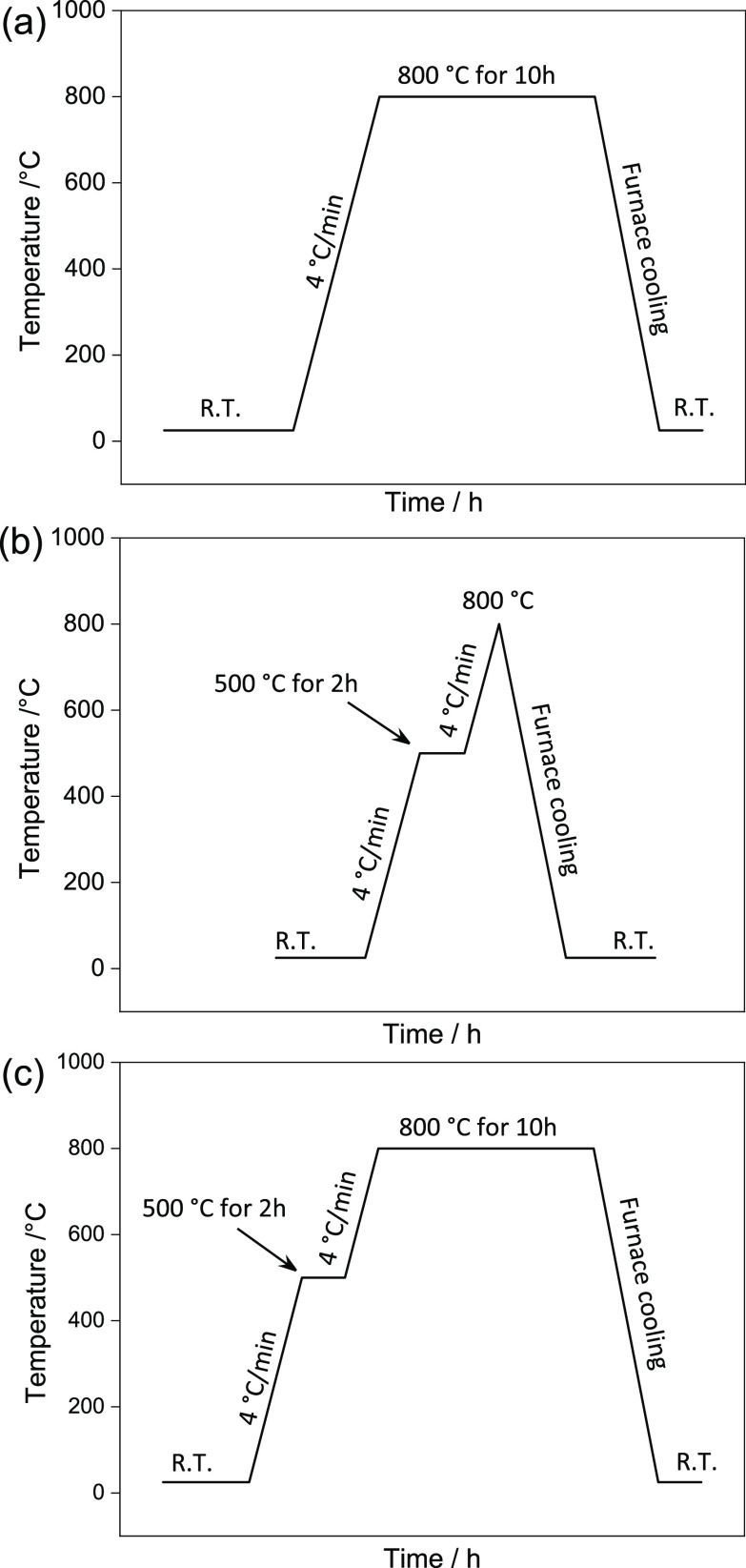
Heating program (a) with only one holding step at 800 °C for 10 h; (b) with only a preannealing step at 500 °C for 2 h; and (c) with both a holding and a preannealing step [combination of (a,b)].

The NCM precursors (NCM811: Ni_0.8_Co_0.1_Mn_0.1_(OH)_2_ and NCM111: Ni_1/3_Co_1/3_Mn_1/3_(OH)_2_) are synthesized by coprecipitation. A 1.5 M aqueous solution of metal ions consisting of NiSO_4_·6H_2_O (Acros Organics, purity: ≥99%), CoSO_4_·7H_2_O (Acros Organics, purity: ≥99%), and MnSO_4_·H_2_O (Carl Roth, purity: ≥99%) in an 8:1:1 (Ni_0.8_Co_0.1_Mn_0.1_(OH)_2_) or 1:1:1 (Ni_1/3_Co_1/3_Mn_1/3_(OH)_2_) ratio, a 12 wt % ammonia solution (Acros Organics, purity: ≥99%), and a 4.875 M sodium hydroxide solution (Fischer Chemical, purity: 99.44%) were separately and simultaneously pumped into a three-necked flask at a rate of 0.5, 0.3, and 0.25 mL min^–1^, respectively. The pH of the mixture is controlled between 10.9 and 11.1 with the manual addition of 4.875 M sodium hydroxide. The reactants are stirred for 4 h at 60 °C under an N_2_ atmosphere with a stirring speed of 1000 rpm. Then, the precipitates are filtered, washed, and dried at 80 °C for 24 h. A Brunauer–Emmett–Teller (BET) and a particle size analysis were performed. The two precursors do not show significant differences in surface area and particle size distribution (Figure S1). To obtain the final rhombohedral lithium transition-metal oxides NCM111 (LiNi_1/3_Co_1/3_Mn_1/3_O_2_) and NCM811 (LiNi_0.8_Co_0.1_Mn_0.2_O_2_), the precursors are calcined with LiOH·H_2_O (Fischer Chemical, purity: ≥99%, contains carbonate impurities; the origin of carbonate impurities upon the synthesis is discussed in the Supporting Information, Figures S2 and S3) at a molar ratio of 1:1.03. In the case of NCM811, O_2_ was flushed through the tube upon calcination, while for NCM111, the tube was left open to the air. The samples are subjected to different heating programs (Table 1 and Figure 1) and are labeled accordingly. The final sintering temperature of 800 °C was determined in a precalcination experiment using the NCM111 precursor/LiOH·H_2_O mixture and heating it to 1000 °C (Figure S4). The choice of the preannealing step is discussed in the Supporting Information (Figure S5).

**Table 1:** Samples with Different Heating Programs (with: √; without: ×)

	heating program		
precursor	preannealing at 500 °C for 2 h	holding at 800 °C for 10 h	sample name
NCM811-OH	×	√	Hold-NCM811
NCM811-OH	√	×	Pre-NCM811
NCM811-OH	√	√	Comb-NCM811
NCM111-OH	×	√	Hold-NCM111
NCM111-OH	√	×	Pre-NCM111
NCM111-OH	√	√	Comb-NCM111

The synthesis of NCM811 and NCM111 was studied using three different calcination programs as described in Table 1 and Figure 1: this includes (i) heating the mixture of the precursor and LiOH·H_2_O directly to 800 °C and annealing it for 10 h (samples are labeled as Hold-), (ii) preannealing the mixtures at 500 °C for 2 h before heating to 800 °C (label: Pre-), and (iii) a combination of the first two programs with a holding step at 500 and 800 °C (label: Comb-). The precursors (labeled as −OH) are obtained *via* coprecipitation as described in the Experimental Section.

### 2.2 Sample Characterization

The phase purity of the different NCMs is confirmed by powder diffraction measurements using a Bruker D8 ADVANCE X-ray diffractometer (Cu Kα_1,2_, λ(Kα_1_) = 1.54060 Å, λ(Kα_2_) = 1.54443 Å). The morphology of the synthesized precursors and NCMs was examined using scanning electron microscopy (SEM, Carl Zeiss AURIGA, Carl Zeiss Microscopy GmbH) at an accelerating voltage of 3.0 kV and a working distance of 3 mm. The thermal stabilities of the precursors were investigated by thermogravimetric analysis (TGA) (Thermogravimetric Analyzer Q5000 IR) for which the precursors were heated to 800 °C with a heating rate of 4 °C/min in air. BET measurements (nitrogen adsorption/desorption) were conducted using a Micromeritics ASAP 2020 instrument after degassing the samples at 200 °C for 6 h. A particle size analysis was performed using a CILAS particle size analyzer.

### 2.3 Electrochemical Characterization

Electrode preparations were performed using 80 wt % of the NCMs, 10 wt % C65 (Super C65, Imerys Graphite & Carbon), 10 wt % polyvinylidene difluoride (Kynar flex), and *N*-methyl pyrrolidone (Sigma-Aldrich, 99.5%) as the processing solvent. The electrode paste was cast onto an Al-foil (20 μm thickness, Goodfellow) with a doctor blade (gap height 200 μm, ZAF 2010, Zehntner). The wet film was dried at 80 °C overnight in an oven. 12 mm diameter samples were punched out of the dried electrodes and assembled in a dry room into 2032-coin cells with two layers of celgard2500 (16 mm Ø), 35 μL of LP572 [1 M LiPF_6_ in ethylene carbonate/ethyl methyl carbonate, 3:7 by weight, with 2 wt % vinylene carbonate, BASF, battery grade], and metallic lithium as a counter electrode (15 mm Ø, Albemarle, battery grade). The as-prepared samples’ rate handling and cycling stability were measured using a Maccor Series 4000 automated test system at 20 °C, applying a voltage of 3.0–4.3 V *versus* Li^+^/Li. For NCM111, 160 mA h/g, and NCM811, 180 mA h/g, respectively, were used as the nominal capacity to calculate the C-rates.

### 2.4 *In Situ* X-ray Powder Diffraction

*In situ* SXPD, performed at Beamline I11, Diamond Light Source UK (λ = 0.826556(2) for NCM811 and 0.826562(2) Å for NCM111, calibrated with NIST SRM Si640c)^34^ and the powder diffraction beamline at ANSTO, Au, (λ = 0.727140(1) Å for NCM111, calibrated with NIST SRM Si640d),^35^ as well as a laboratory X-ray powder diffraction instrument (LXRD, Rigaku SmartLab SE 3 kW, Mo Kα with λ = 0.7107 Å) were used to study the calcination of NCM111 and NCM811. The experimental setup is depicted in Figure S6. The temperature was calibrated with a Pt standard within the capillary (1 mm Ø).

The SXPD and LXRD patterns were analyzed using the software package Fullprof (for more details, refer to the section XRD pattern analysis).^36,37^ The Ni/Li disorder was calculated from Rietveld refinements (the refined occupancy of Ni in the Li 3b sites/amount of 3a sites * 100%). The Ni/Co/Mn ratios were fixed at their expected molar ratios (0.33:0.33:0.33 for NCM111 and 0.8:0.1:0.1 for NCM811). The structural input parameters for the present phases were obtained from crystallographic data files (Table S1).^38−45^ Typical XRD patterns for the NCMs and the Rietveld refinement are plotted in the Supporting Information (Figure S7 for NCM111s, Figure S8 for NCM811s, and Rietveld parameters in Table S2).

In the subsequent discussion, reflections and planes are marked with acronyms of their space group, for example, 003 reflection (*R*3̅*m*) is labeled as *R*003, 003 plane (*R*3̅*m*) as *R*(003), 001 reflection (*P*3̅*m*1) as *P*001, 001 plane (*P*3̅*m*1) as *P*(001), 111 reflection (*Fm*3̅*m*) as *F*111, and 111 plane (*Fm*3̅*m*) as *F*(111).

### 2.5 Near X-ray Absorption Fine Structure Spectroscopy

All NCM111 and 811 samples were collected during the heating procedure. The samples were taken out of the furnace when the set temperature was reached and quenched in liquid nitrogen. NEXAFS measurements were performed at IQMT’s soft X-ray beamline WERA at the Karlsruhe synchrotron light source KARA (Germany). NEXAFS measurements at the Ni L_2,3_, Co L_2,3_, Mn L_2,3_, and O K were carried out in fluorescence yield (FY) detection mode. The photon-energy resolution in the spectra was set to 0.2–0.4 eV. Energy calibration (using a NiO reference), dark current subtraction, division by *I*_0_, background subtraction, data normalization, and absorption correction were performed as described in refs (46) and (47).

## 3 Results and Discussion

### 3.1 Material Characterization

**Figure 2:**
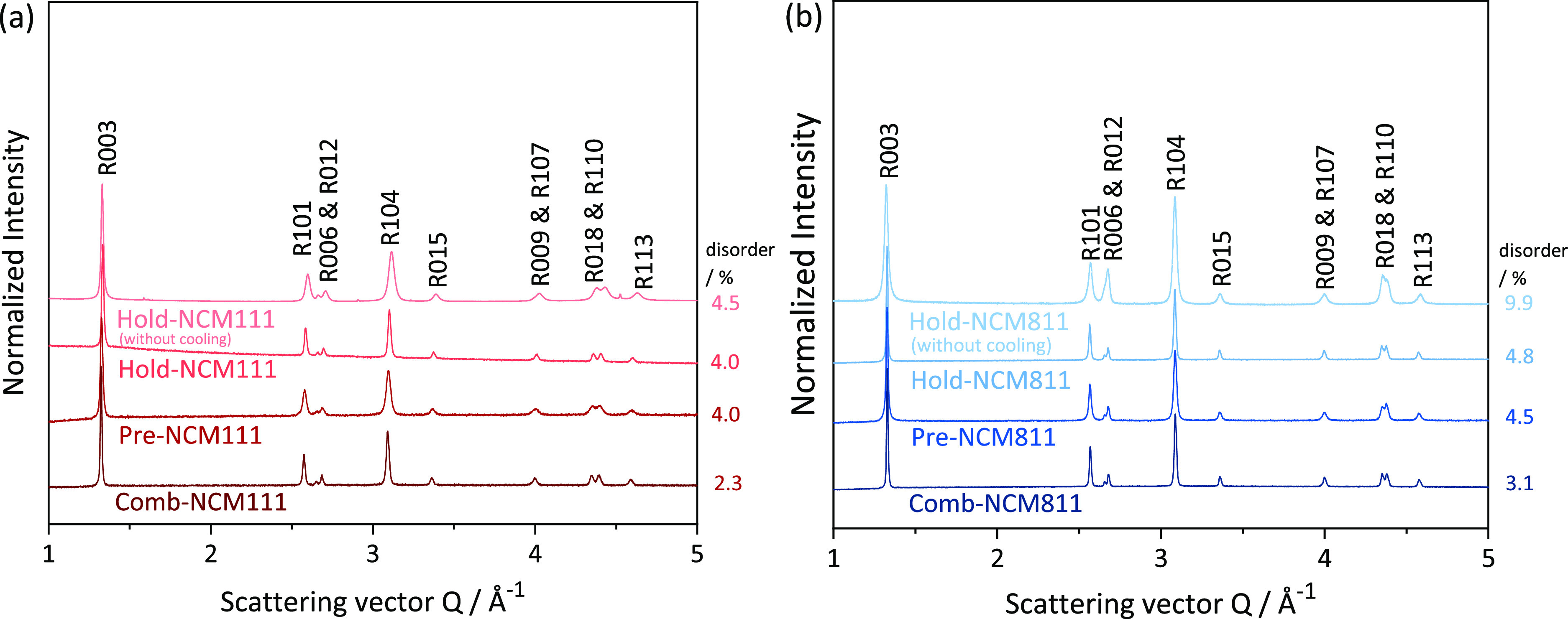
XRD patterns of (A) NCM111s and (B) NCM811 synthesized through different heating treatments with the corresponding disorder/%. XRD patterns of Hold-, Pre-, and Comb-NCMs are obtained from LXRD and XRD patterns of Hold-NCMs without cooling from SXPD.

The *ex situ* LXRD patterns of all NCMs and the refined disorder are compared in Figure 2. All samples can be indexed with a NaFeO_2_ hexagonal structure (*R*3̅*m* space group) and are phase-pure. In all cases, the cation mixing in NCM111 is lower than in NCM811. Moreover, the Me/Li disorder of the Pre- and Hold-NCMs is always higher than that of the Comb-NCMs. Preannealing at 500 °C for 2 h and holding at 800 °C for 10 h can lower cation mixing. The splitting of the reflection doublets *R*006/*R*012 and *R*018/*R*110 (Figure S9 show their crystallographic planes) indicates a highly ordered layered structure with a relatively large crystallite size (see Figure S7c).^48^ Pre-NCMs have broader reflections and incompletely separated reflection pairs compared to Hold- and Comb-NCMs, meaning that the holding step at high temperatures is significant to the formation of well-ordered layered structures. Note that the disorder also decreases upon the final cooling step, Figure 2 (samples without cooling). This was also confirmed by comparing the final samples with a quenched sample quenched in liquid nitrogen directly after calcination (Table S3). The higher disorder at the end of calcination compared to the final value (Figure 2) might also be correlated with larger Debye–Waller factors which are known to correlate with site occupancies. The morphologies of the as-prepared precursors and NCMs are shown in Figure S10. Figure S11 shows the thermal stabilities of these two precursors. NCM111OH reveals a better thermal resistance compared to NCM811OH.

**Figure 3:**
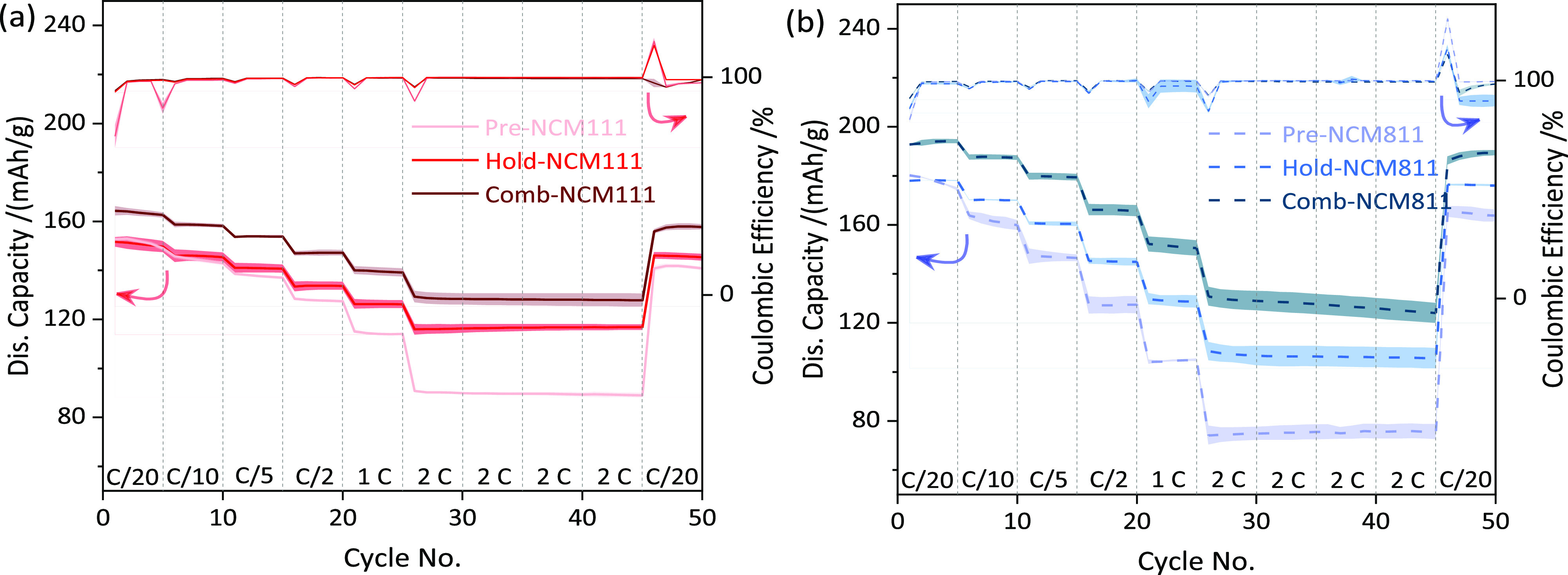
Rate handling performance of different NCMs. (a) NCM111. (b) NCM811.

The corresponding cycling data of the different NCMs are gathered in Figure 3. The dQ/dV and capacity retention as a function of the C-rate of all synthesized samples are given in Figure S12. The first discharge capacities of the NCM811s (Comb-NCM811: ∼193 mA h/g, Pre-NCM811: ∼180 mA h/g, Hold-NCM811: ∼178 mA h/g) are higher than those of the NCM111s (Comb-NCM111: ∼164 mA h/g, Pre-NCM111: ∼153 mA h/g, Hold-NCM111: ∼152 mA h/g). The performance of the materials (especially if both, a preannealing and a final annealing step are applied) is comparable to the literature.^49−51^ However, with an increasing C-rate, the specific capacity of NCM811 decreases by about 30 mA h g^–1^ (Comb-NCM811) to 100 mA h g^–1^ (Hold-NCM811) from C/20 to 2C while the NCM111 capacity decreases about 15 to 50 mA h g^–1^ (Comb- and Hold-NCM111, respectively), indicating that Ni-poor materials have a better rate capability. Moreover, Comb-NCMs demonstrate better rate capabilities than Pre- and Hold-NCMs. The NCM811s reveal a higher disorder than the NCM111s (Figure 2), and Comb-NCMs have a lower disorder than Pre- and Hold-NCMs, which can be one of the reasons for better rate-dependent performance. From the electrochemical characterization, it is evident that both the preannealing and the 800 °C holding step are beneficial for obtaining a well-ordered layered structure with good cycling performance.

### 3.2 Calcination: 800 °C Holding Step

**Figure 4:**
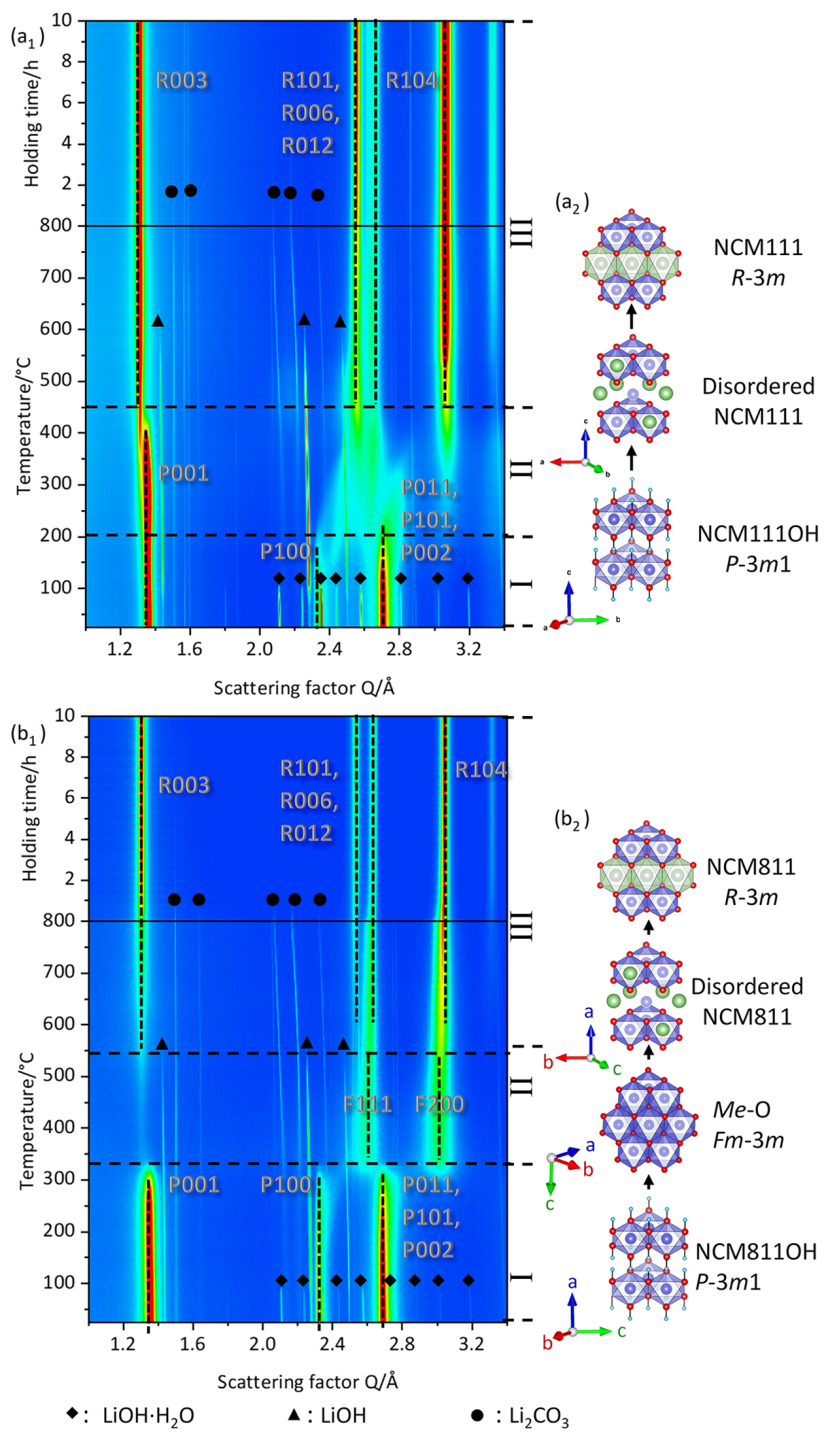
*In situ* SXPD in contour plot of NCM111 (a_1_) and NCM811 (b_1_), collected upon calcination without preannealing; (a_2_,b_2_) structural transformation during the synthesis of NCM111: atoms in green (Li), blue (transition metal), red (O), and light blue (H). On the right side, the structural changes upon calcination are depicted.

The structural origin of the differences is further investigated by performing the calcination with a holding step at 800 °C holding for 10 h, with a pre-annealing step at 500 °C for 2 h, and a combination of both (see Figure 1). *In situ* SXPD patterns of the calcination of NCM111 and NCM811 with an 800 °C holding step are shown in Figure 4a_1_,b_1_. The schematic phase transformations revealed with Rietveld refinement are depicted in Figure 4a_2_,b_2_.

**Figure 5:**
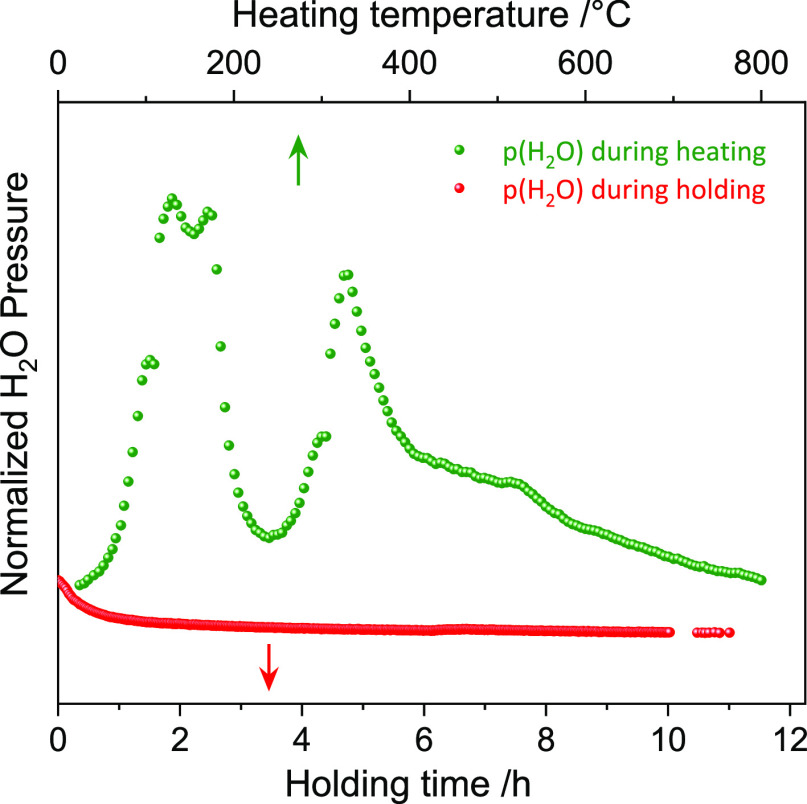
MS data show the H_2_O pressure *vs* temperature obtained during the synthesis of NCM811. MS data for the synthesis of NCM111 can be found in Figure S13. Since the capillary was left open to air, the H_2_O signal from the NCM111OH material is obscured because of the existence of H_2_O in the air.

**Figure 6:**
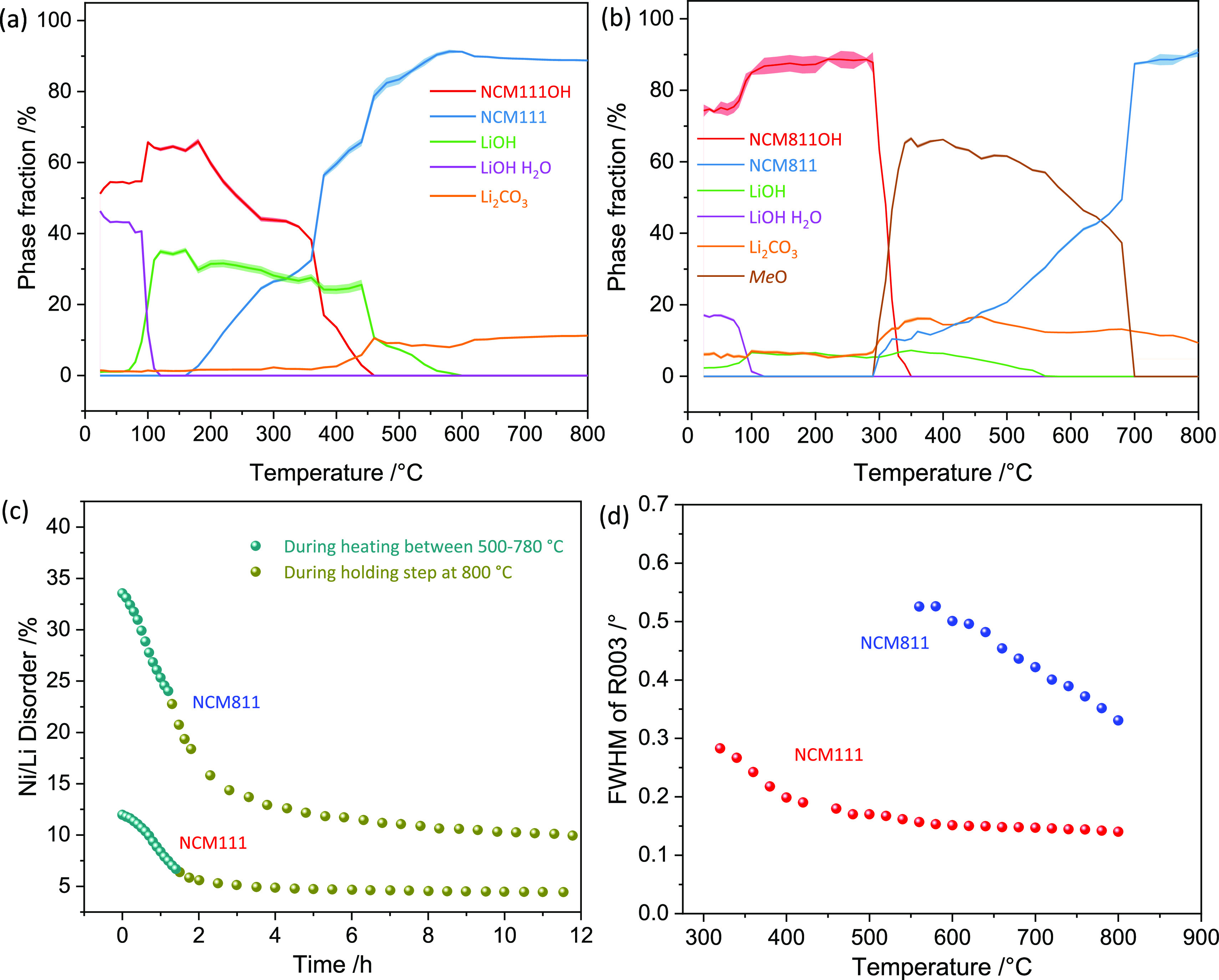
Phase fraction evolution as a function of temperature for (a) NCM111, (b) NCM811, and (c) Ni/Li disorder against the time in hours from 500 °C to the end of the holding step, and (d) FWHM of the *R*003 reflection changes over temperature.

The calcination process can be divided into three stages. Upon stage I (RT to ∼200 °C), the precursors (NCM111-OH and NCM811-OH) remain unchanged, but the LiOH·H_2_O reflections disappear at >100 °C (diamonds), accompanied by the appearance of LiOH reflections (triangles) due to entrapped water loss (Figure 4). This agrees with the 1st H_2_O peak in the MS data of NCM811, Figure 5. Note that the precursors might also lose water because they are only dried overnight at 80 °C before calcination. This water loss probably can be attributed to the second peak in Figure 5. The phase content of NCM111-OH/NCM811-OH, LiOH·H_2_O, LiOH, intermediate MeO phases of the precursor, and Li_2_CO_3_ upon the calcination is shown in Figure 6a,b. Stage II marks the onset of the changes in the precursors, while in stage III, the rhombohedral structure appears. Note that the temperatures at which these structural rearrangements happen are pretty different for NCM111 and NCM811. In the case of NCM111, stage II is found between ∼200 and 450 °C. The *P*100, *P*011, and *P*101 NCM11-OH reflections vanish at ∼200 °C, while other reflections, such as the *P*001, are present until 360 °C. This can be explained by water loss from the precursor because hydrogen and oxygen sit in the *P*(100), *P*(011), and *P*(101) planes (OH 2d sites). The *P*001 reflection represents the *d*-spacing between the transition-metal layers, and the presence of this reflection until 360 °C shows that the layered structure remains intact. The reflections *R*003 of the final rhombohedral structure and *P*001 share the same crystallographic plane (transition metal plane), and thus, the transformation of the *P*3̅*m*1 into the *R*3̅*m* does not require a significant rearrangement of atoms.

In the case of NCM811, the first changes in the precursor reflections are found at 325 °C (onset of stage II), Figure 4b_1_. In contrast to NCM111-OH, for which the layered structure remains intact, almost all NCM811-OH reflections become very broad and weak in intensity up to that they vanish; that is, NCM811-OH transforms almost entirely into cubic MeO_*x*_ (rock salt-type oxide, *x* ≈ 1). The structural collapse is evident from the disappearance of the precursor reflections and the appearance of new reflections such as *F*111 and *F*200 (Figure 4b_1_) and is confirmed by Rietveld refinement, Figure 6b. The phase transformation is accompanied by a third H_2_O peak in the MS spectrum (Figure 5), which means that almost all protons that isolate the adjacent transition-metal layers in the *P*3̅*m*1 structure (NCM811-OH) are removed. Due to the repulsive interactions between the remaining O atoms in the adjacent layers, transition metals migrate into the interslabs to stabilize the structure forming the new cubic phase (*Fm*3̅*m*). In this intermediate phase, the arrangement of the transition metals in alternating layers gets lost (Figure 4b_1_, stage II), which is, for example, evident from the small and broad *P*001 reflection which even remains after 325 °C, Figure S7b. Note that the decomposition of NCM811-OH is at lower temperatures (∼200 °C) than for NCM111-OH (∼300 °C), as deduced from *in situ* SXPD. The *P*001 reflection of NCM111-OH withstands up to 400 °C, indicating that the Me layers between those Li ions can be incorporated and remain stable until enough Li ions are present to fill the interstitial layer. In contrast, the Me layer disappears in the case of NCM811-OH at ∼325 °C, Figure 4b_1_ (*e.g.*, the disappearance of the *P*001 reflection), which makes energy-consuming phase transformations necessary to restore the layered structure upon Li intercalation.

At around 400–450 °C, the reflections of the final NCM111 structure (*e.g.*, *R*003 and *R*104) are visible for the first time (Figure 4a_1_), marking the onset of stage III. The increase of the layered structure is at the expense of NCM111-OH and LiOH, significantly above the melting point of LiOH (446 °C), verified by the refinement of the phase contents, Figure 6a. This suggests that a massive Li insertion into the host structure takes place once the melting point of LiOH is reached. Thereby, the NCM111-OH precursor (*P*3̅*m*1, herein depicted in trigonal setting) does not entirely decompose, but with water removal and subsequent Li intercalation, the rhombohedral structure (*R*3̅*m*) appears. Note that trigonal and rhombohedral settings are different depictions of the same structure using different coordinate systems. The *P*(001) plane of NCM111OH and the *R*(003) plane of the layered NCM111 structure result from the same transition-metal layer in the host structure representing a close planar *d*-spacing (see the transformation matrix in eq 1 as follows and Table 2).
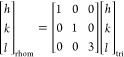
1where rhom is short for the rhombohedral structure (*R*3̅*m*), tri for the trigonal structure, and (*h k l*) for the crystallographic plane. Due to the larger radii of lithium ions, the elongation of the *c* lattice [and thus the sudden jump of the reflection *P*(001)/*R*(003)] at ≈450 °C is induced with Li intercalation.

**Table 2:** Crystallographic Planes of the Trigonal and Rhombohedral Structures

crystallographic planes in the trigonal	corresponding planes in the rhombohedral
*P*(001)	*R*(003)
*P*(002)	*R*(006)
*P*(110)	*R*(110)
...	...

Between 450 and 800 °C (stage III), a highly defective rhombohedral NCM111 structure gradually develops and perfects over time, Figure 4a1. The rhombohedral reflections continuously increase in intensity (Figures 4a1^6^), while the Li/Ni disorder and the full-width at half-maximum (FWHM) of the *R*(003) reflection decrease, Figure 6c,d. The observed decrease in the FWHM has its origin in increasing crystallite size and decreasing microstrain due to an ongoing ordering process.^52^ Due to the difference in electron density in the layered structure (Figure. S14), *R*003 (only Me 3b sites) increases in intensity, while the *R*104 (Me 3b, Me 3a, and O 6c sites) intensity remains constant upon lattice perfection.

At high temperatures, the kinetic energy of the atoms increases, which accelerates ion mobilities. Therefore, defects such as cation vacancies and antisites are annihilated faster. The disorder decreases from ∼12 to ∼6.5% as the temperature increases from 500 to 800 °C, Figure 6c. Once the temperature reaches 800 °C, the reflection pairs *R*006/*R*012 of NCM111 start to split (Figure S7c), suggesting a highly crystalline form of NCM111.^53^ During the holding step at 800 °C, the degree of the disorder tends toward a constant value of ≈5%, indicating slower ordering kinetics. Note that the disorder is higher compared to the final sample at room temperature (Figure 2 and Table S3), which means that the subsequent cooling of the sample further facilitates the ordering process. Moreover, a Li_2_CO_3_ impurity (circles in Figure 4), which arises from the impurities in the raw material (Figure S2), is apparent throughout the synthesis. The Li carbonate is a detrimental inclusion in battery cathode materials, and preventing this undesirable phase formation should be a promising direction for future process improvement.

The evolution of the NCM811 structure is more of a continuous process than a sudden phase transformation. As the temperature increases beyond 325 °C upon the synthesis of NCM811, the *F*111 and *F*200 reflections slightly shift to higher *Q*-values, which can be attributed to Li intercalation into the cubic structure (MeO_*x*_ → Li_*y*_MeO_*x*_, 1 ≤ *x* ≤ 2, 0 < *y* ≤ 1). The shifts indicate that the cubic structure exhibits negative expansivity. The reflections of the rhombohedral phase (*e.g.*, *R*104) emerge at ∼450 °C, marking stage III’s onset. However, the *R*104 reflection is initially extensive due to a high Li/Ni disorder (Figure 6c), and the *F*200 reflection superimposes it. According to the transformation matrix from the cubic to the rhombohedral structure in eq 2 as follows^24^
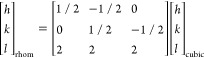
2where rhom is short for the rhombohedral structure (*R*3̅*m*), cubic for the cubic structure. (*Fm*3̅*m*), and (*h k l*) for the crystallographic plane; some crystallographic planes bore from the previous host structure, which is listed in Table 3. The *F*200 and *R*104 reflections share the same planes. Although *R*104 shows a slightly higher *Q*-value than the *F*200, it is not possible to distinguish one phase from the other by refinement. However, at 500 °C, the refinement significantly improves by adding two transition-metal oxide phases, while at 800 °C, one phase refinement (*R*3̅*m*) is more suitable; see Figure S15. Initially, reflections that are transferred from the NiO phase into the *R*3̅*m* phase appear broader than reflections introduced solely due to the separation of Ni and Li into different layers. The rock salt-type structure was omitted in the refinements at temperatures > 700 °C because the reflection broadenings of reflections with both contributions (*Fm*3̅*m* and *R*3̅*m*) and only the *R*3̅*m* contribution follows, respectively, a single Williamson Hall line (see Figures S15 and S16).

**Table 3:** Crystallographic Planes of the Rock Salt-Type and Rhombohedral Structures

crystallographic planes in the rock salt	corresponding planes in the rhombohedral
*F*(111)	*R*(006)
*F*(200)	*R*(104)
*F*(220)	*R*(018)
...	...

Stage III (∼540 to 800 °C) and the subsequent holding step upon the synthesis of NCM811 are similar to NCM111. The reflections of the rhombohedral phase increase and sharpen, as indicated by the FWHM evolution of *R*003 (Figure 6d and Table S4). The ongoing temperature rise facilitates the ordering process, and the cation disorder keeps decreasing to a constant value of 10% (Figure 6c). In contrast to NCM111, the *R*006/*R*012 reflection pair has not been separated by the end of stage III (the end of the holding step). Instead, the splitting of these reflections begins upon the final cooling, much later than for NCM111; see Figures S7c and S8c. This suggests that due to the existence of the intermediate rock salt-type structure MeO_*x*_ NCM811 needs more time (and/or energy) to become a well-ordered layered structure with a relatively large crystallite size.

### 3.3 Calcination: 500 °C Preannealing Step

**Figure 7:**
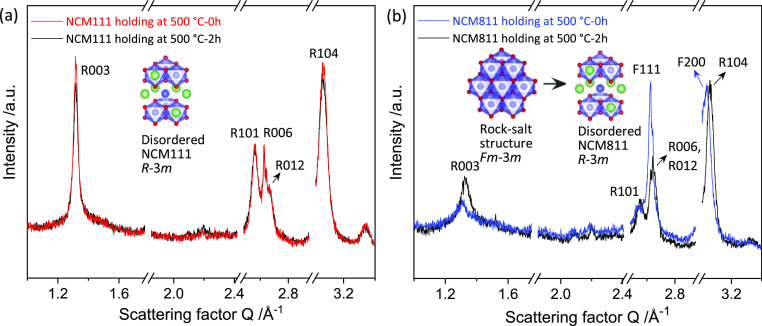
Comparison of the *in situ* lab XRD patterns before and after preannealing: (A) NCM111; (B) NCM811; the excluded regions cover some reflections of the alumina sample holder.

Preannealing at 450 to 550 °C before the real annealing step at 800 °C is adopted in many works^54,55^*via* different preparation processes, such as sol–gel,^56^ coprecipitation,^57^ and spray-drying.^58^ To unveil the reason for this pretreatment, preannealing of NCM111 and NCM811 at 500 °C for 2 h was monitored *via in situ* lab XRD (Mo Kα source). The collected data is plotted in Figures 7 and S17. During preannealing, all NCM111 reflections in Figure 7a can be assigned to the rhombohedral structure, and the *R*003 reflection increases in intensity over time, whereas significant deviations from this behavior are observed for NCM811, Figure 7b. First, some reflections associated with the rhombohedral structure (*R*006, *R*012, and *R*104) appear at the expense of the reflections belonging to the rock salt structure such as *F*111 and *F*200. As mentioned above, the *R*(006) and the *R*(104) planes of the rhombohedral structure maintain the same parent planes of the *F*(111) and *F*(200) planes (rock salt structure and Table 3). This explains that the *R*003 and *R*101 reflections of NCM811 are much broader compared to the corresponding NCM111 reflections. Moreover, although *R*003 (NCM811, Figure 7b) gets more visible over time upon the holding step at 500 °C, its intensity is not comparable to the *R*003 reflection of NCM111, indicating that Me/Li separation into alternating O-layers takes place later in NCM811. Nevertheless, in both cases (NCM811 and NCM111), the additional holding step at temperatures above the melting point of LiOH lowers cation mixing. In addition, it promotes the phase transformation from the cubic (*Fm*3̅*m*) to the rhombohedral (*R*3̅*m*) structure in NCM811.

**Figure 8:**
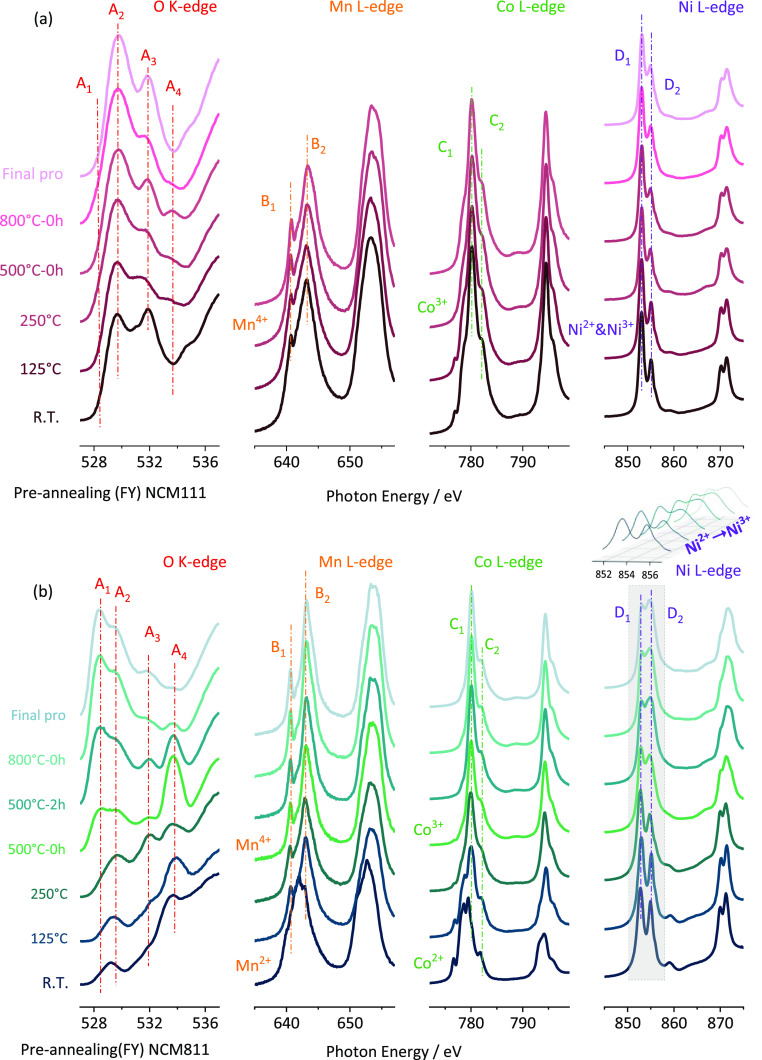
NEXAFS spectra of O K edge, Mn L edge, Co L edge, and Ni L edge for (a) NCM111s and (b) NCM811s at different temperatures.

**Figure 9:**
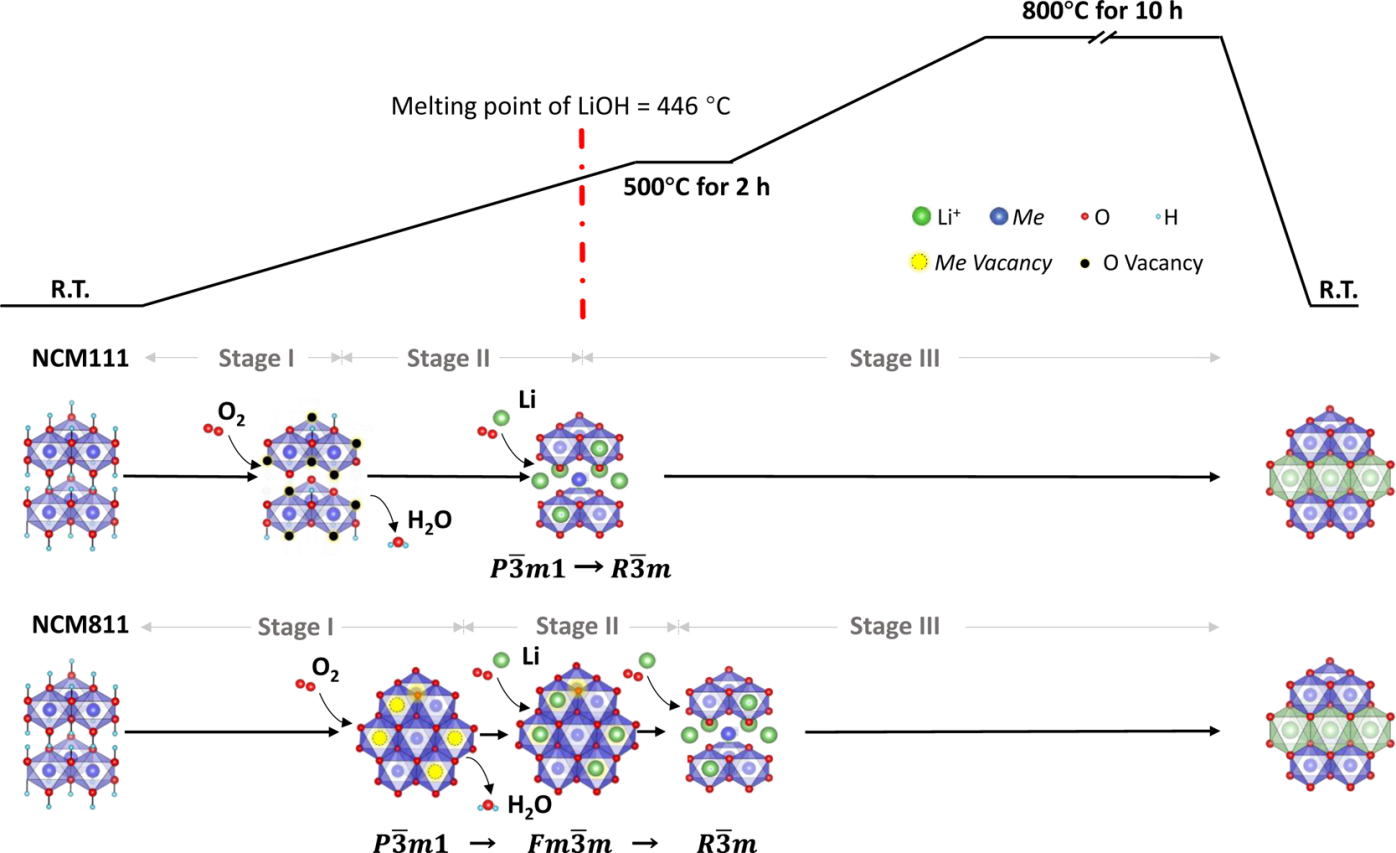
Schematic representation of the reaction pathways for NCM111 and NCM811.

Including the preannealing step in the calcination process benefits the overall structural evolution in both cases. Me/Li disorder is reduced by 1.7% (both NCM111 and NCM111), including the holding step at 500 °C, Figure 2. However, to understand the origin of the different structural evolution of NCM111 and NCM811, the oxidation of the transition metals upon Li intercalation can be monitored using near-edge X-ray absorption fine structure spectroscopy (NEXAFS) in Figure 7. Here, we use the FY, which is a more bulk-sensitive signal. Co and Mn oxidation are complete at temperatures > 250 °C in the case of NCM111 (Figure 8a, peaks B_1_, B_2_, C_1_, and C_2_). To keep the charge neutrality in the rhombohedral structure, the mean oxidation state of the transition metals is +3. Co reveals a +3-oxidation state in the final rhombohedral structure, while Mn is oxidized to +4.^26^ In the case of NCM111, which has the same amount of Ni and Mn, this means that Ni can remain in a +2 oxidation state as deduced in Figure 8b, the preferred d8 configuration of Ni. In contrast, the amount of Ni in NCM811 is 4 times the amount of Mn. Thus, Ni (initially in a +2 configuration) needs to be partially oxidized to a +3 configuration (Figure 8, last column, upcoming peak at 856 eV, peak D_2_). This oxidation takes place at temperatures > 500 °C and is accompanied by Ni–O hybridization (Figure 8, O K edge, peaks at 528 and 530 eV, A_1_ and A_2_). The holding step at this temperature is therefore required to oxidize Ni completely. If a preholding step is applied for 2 h, the oxidation of Ni is more complete at 800 °C (Figures 9 and S18).

## 4 Reaction Pathways

NCM111 and NCM811, as representatives for Ni-poor and Ni-rich NCMs, show two different reaction pathways upon calcination. Hydroxy groups from the NCM111-OH are removed above 200 °C (Figures 4a_1_ and 9), generating oxygen and hydrogen vacancies for the lithiation process (stage I). This is visible by the broadening of the *P*-reflections containing a contribution in the direction of the transition-metal planes (*e.g.*, *P*100, *P*002, *P*101, and *P*011). In contrast, the transition-metal layers (*e.g.*, the *P*001 reflection) remain intact up to ∼450 °C, Figure 4a_1_. When the melting point of LiOH (446 °C) is reached (stage III), the lithiation of the OH-defect precursor structure is promoted, indicated by the disappearance of all precursor reflections and the occurrence of (or the transformation into) the *R*-reflections of the final rhombohedral phase.

The NCM811-OH decomposes at ∼325 °C, including a collapse of the Me layers since all *P*-reflections disappear (Figures 4b_1_ and 9). In contrast to NCM111-OH, the structure transforms into a cubic MeO rock salt structure (and a minor content, <2%) in which the Me (and Me vacancies) randomly occupy the transition-metal and interlayer octahedral sites (Figure 9). This comes along with water loss (Figure 4b_1_) and agrees roughly with the onset of the decomposition temperature of the NCM811-OH at >241 °C. The cubic structure is the thermodynamically preferred structure of Ni-rich MeO (the structure with the highest lattice energy) in the absence of Li ions. Thus the high Ni content drives the transformation. Nevertheless, once the melting point of LiOH is reached (446 °C), Li^+^ is incorporated into the Me–O framework (stage II). The oxidation of the transition metals to higher valence states leads to the formation of cation vacancies,^59,60^ which is the driving force for the lithiation. The subsequent preannealing at 500 °C above the melting point of LiOH helps further promote the lithiation into the intermediate cubic structure. Once a sufficient amount of lithium ions is distributed over the cubic structure, the segregation of lithium ions and transition metals starts due to their difference in radius, similar to the synthesis of LiNiO_2_.^24^ Upon the 2 h holding step at 500 °C (preannealing, Figure 9), the elongation of the *c* lattice parameter and a rhombohedral distortion, all driven by lowering steric constraints, lead to the new *R*3̅*m* rhombohedral structure. Upon the subsequent annealing, the number of defects in the rhombohedral structure decreases, as is observed for NCM111. For the calcination of NCM111, the 2 h holding step at 500 °C lowers the number of defects but is not as essential as in the case of NCM811 since the separation of Me layers in the *c*-direction stays stable upon the entire synthesis and Ni^2+^ oxidation, which takes place at 500 °C, is not necessary.

The melting point of LiOH plays a vital role in the synthesis of both NCM111 and NCM811. It offers sufficient lithium ions for Li incorporation into the Me–O framework and facilitates the lithiation process. The synthesis differs in the different thermal resistances. NCM811-OH undergoes a phase transformation into a rock salt structure before reaching the melting point of LiOH. The subsequent separation of Li ions and Me, as well as the oxidation of Ni^2+^ to Ni^3+^, requires more time and energy compared to NCM111, for which the Me layers remain stable upon the entire calcination, and Ni remains in a +2 oxidation state. This explains the need for pure oxygen versus air in the case of NCM811 (*vs* NCM111) and the necessity of the holding step, which is to promote Me and Li separation back to a layered structure as well as facilitate Ni oxidation. A stronger oxidizing atmosphere can kinetically accelerate the oxidation of transition-metal ions and the creation of vacancies, thus improving the reaction rate for Li intercalation. Additionally, the preannealing step over the melting point of LiOH supports the phase transformation.

Higher temperatures can accelerate the reaction kinetics as well. Still, at temperatures > 800 °C (for NCM111), not only the ordering process is accelerated but also lithium loss speeds up, which leads to Li vacancies (3b site) near the surface.^61^ Then, the adjacent Ni ions migrate into these Li vacancies, leading to a rise in cation mixing. Therefore, a suitable annealing temperature is required for the calcination of NCMs. Besides the decreasing defects upon subsequent heating from the 2 h holding step (500 °C) onward, the final cooling to room temperature further increases Me/Li separation.

Furthermore, after long-term cycling, Ni-rich layered oxides undergo lithium loss and oxygen release, especially near the grain boundaries.^29−31^ Thereby, the same rock-salt phase is formed which is observed upon the synthesis of NCM811 around 500 °C. This work shows how the intercalation of Li ions into a rock salt structure proceeds. In detail, it reveals that the oxidation of Ni^2+^ in the rock salt structure mainly takes place at ∼500 °C during the synthesis process which makes a preannealing step at this temperature mandatory. At lower temperatures, intercalation cannot take place because of the absence of mobile Li ions as the melting point of LiOH is not reached, while at higher temperatures, it remains incomplete due to the sluggish kinetics of oxidation. The findings suggest that the optimum temperature for Ni oxidation is >446 and <550 °C, which is the best temperature not only for the preannealing step but also for layered oxide cathode material reactivation.

## 5 Conclusions

The synthesis of NCM111 and NCM811 using a 2 h precalcination step at 500 °C and a 10 h holding step at 800 °C upon calcination restricts the cation mixing and improves the electrochemical performance. Differences in the calcination process of Ni-poor (NCM111) and Ni-rich (NCM811) materials mainly manifest in differences in the final disorder (2.3% for NCM111 and 3.1% for NCM811), leading to relatively poor rate capabilities of Ni-rich NCMs. In the case of NCM111, the trigonal precursor (*P*3̅*m*1) transforms into a disordered NCM111 structure at ∼300 °C, and the ordering process reaches an almost constant value above 700 °C. The Me layers of NCM111 remain stable upon the entire calcination which is why Li intercalation for NCM111 happens continuously over 200 °C, leading to constantly changing reflections. In the case of NCM811, the trigonal precursor (*P*3̅*m*1) quickly transforms into a rock-salt structure Li_*x*_Me_1–*x*_O (*T* ∼ 325 to 540 °C) before the melting point of LiOH is reached. At ∼500 °C, the rhombohedral NCM811 evolves (540 to 800 °C), and the Li/Ni disorder gets a constant value at *T* ≥ 800 °C. The preholding step at 500 °C is much more crucial in the case of Ni-rich materials because, at this point, Ni oxidation from Ni^2+^ to Ni^3+^ takes place, which remains incomplete and facilitates disorder if the temperature rises too fast.

The detailed mechanistic understanding of the synthesis of Ni-poor and Ni-rich NCMs is not only crucial for the industrial synthesis of NCMs, but it is also vital considering the circle life of batteries for which the reactivation of cathode materials after extensive cycling and subsequent degradation is similar to the deduced calcination procedure. The reactivation of NCM811 is expected to be more difficult compared to NCM111. Upon degradation of NCMs, a rock salt structure (the same structure formed upon the calcination of Ni-rich NCMs) forms on the surface of the particles. The results show that the higher the Ni content, the more likely a NiO phase forms. Reactivating a NiO structure requires (besides the presence of Li) an oxidizing atmosphere and temperatures above 500 °C. At lower temperatures, even more, NiO will be formed. The present work thus provides onset temperatures for the relithiation of NiO in the presence of Li ions, which can be used as a guide for reactivation studies.

## Supporting Information Available

BET measurements of the NCM111 and NCM811 precursors; refinement of LiOH·H_2_O used upon calcination of the precursors; carbonate reflections upon calcination; *in situ* calcination of NCM111 from RT until 1000 °C; discussion about the choice of the holding step, experimental setup for the *in situ* SXPD study of the calcination process; Rietveld refinements of NCM111 and NCM811 upon calcination; (012), (006), (008), and (110) planes of the rhombohedral structure; SEM images of the precursors and the final products; TGA measurements of the precursors; differential capacity and lifetime of NCM111 and NCM811; MS results (H_2_O signal) upon the calcination of NCM111; (104) and (003) planes of the rhombohedral structure, details about the procedure of Rietveld refinement; and NEXAFS spectra of NCM811 obtained upon calcination without a holding step (PDF)
