# Supplementary material for: Monitoring the Formation of Nickel-Poor and Nickel-Rich Oxide Cathode Materials for Lithium-Ion Batteries with Synchrotron Radiation
Source: Chem Mater. 2023 Jan 31;35(4):1514–26. doi: 10.1021/acs.chemmater.2c02639 (PMC9979376; doi:10.1021/acs.chemmater.2c02639)
Supplement: Supplementary file 1 — cm2c02639_si_001.pdf [file cm2c02639_si_001.pdf]

Supporting information

Monitoring the Formation of Nickel-Poor and Nickel-Rich Oxide Cathode Materials for Lithium-Ion Batteries with Synchrotron Radiation

Bixian Ying[a], Jack R. Fitzpatrick[b], Zhenjie Teng[a], Tianxiang Chen[c], Tsz Woon Benedict Lo[c], Vassilios Siozios[a], Claire A. Murray[d], Helen E. A. Brand[e], Sarah Day[d], Chiu C. Tang[d], Robert S. Weatherup[f], Michael Merz[g,h], Peter Nagel[g,h], Stefan Schuppler[g,h], Martin Winter[a,j], Karin Kleiner[a,]*

[a] *MEET, Battery Research Center, University of Muenster, Corrensstr. 46, 48149 Münster, Germany*

[b] *Imperial College London, Department of Chemistry, Molecular Sciences Research Hub, White City Campus, W12 0BZ, 82 Wood Lane*

[c] *Department of Applied Biology and Chemical Technology, The Hong Kong Polytechnic University, Hunghom, Hong Kong, China*

[d] *Diamond Light Source Ltd, Harwell Science & Innovation Campus, Didcot, Oxfordshire, OX11 0DE, United Kingdom*

[e] *Australian Synchrotron ANSTO, 800 Blackburn Rd., Clayton, VIC 3168, Australia*

[f] *Department of Materials, University of Oxford, Parks Road, Oxford OX1 3PH, United Kingdom*

[g] *Institute for Quantum Materials and Technologies, Karlsruhe Institute of Technology, 76021 Karlsruhe, Germany*

[h] *Karlsruhe Nano Micro Facility (KNMFi), Karlsruhe Institute of Technology (KIT), 76344 Eggenstein-Leopoldshafen, Germany*

[j] *Helmholtz-Institute Münster, Forschungszentrum Jülich GmbH, 48149 Muenster, Germany*

BET and particle size analysis

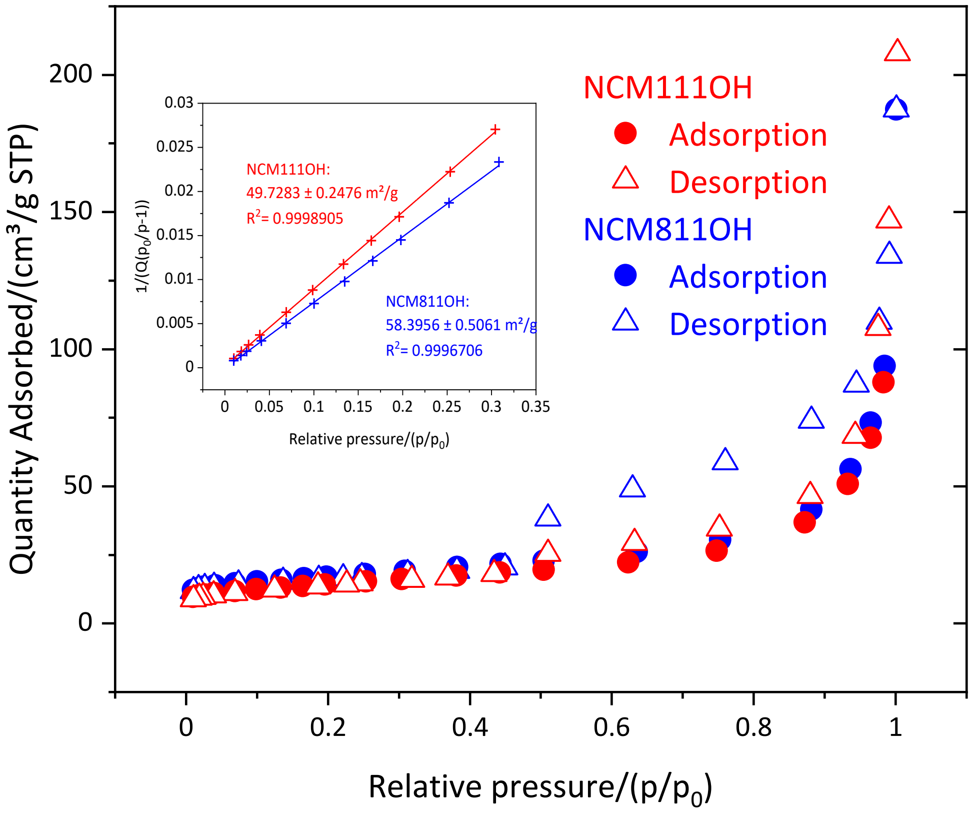

Fig. S1 BET adsorption and desorption curves of the precursors.

According to the BET measurements, the BET surface areas of NCM811OH and NCM111OH are obtained, 58.3956 ± 0.5061 m²/g and 49.7283 ± 0.2476 m²/g, respectively. According to the particle size analysis, D50 values of NCM811O and NCM111OH are 11.14 μm and 13.22 μm, respectively.

Origin of carbonate upon the synthesis of layered oxides

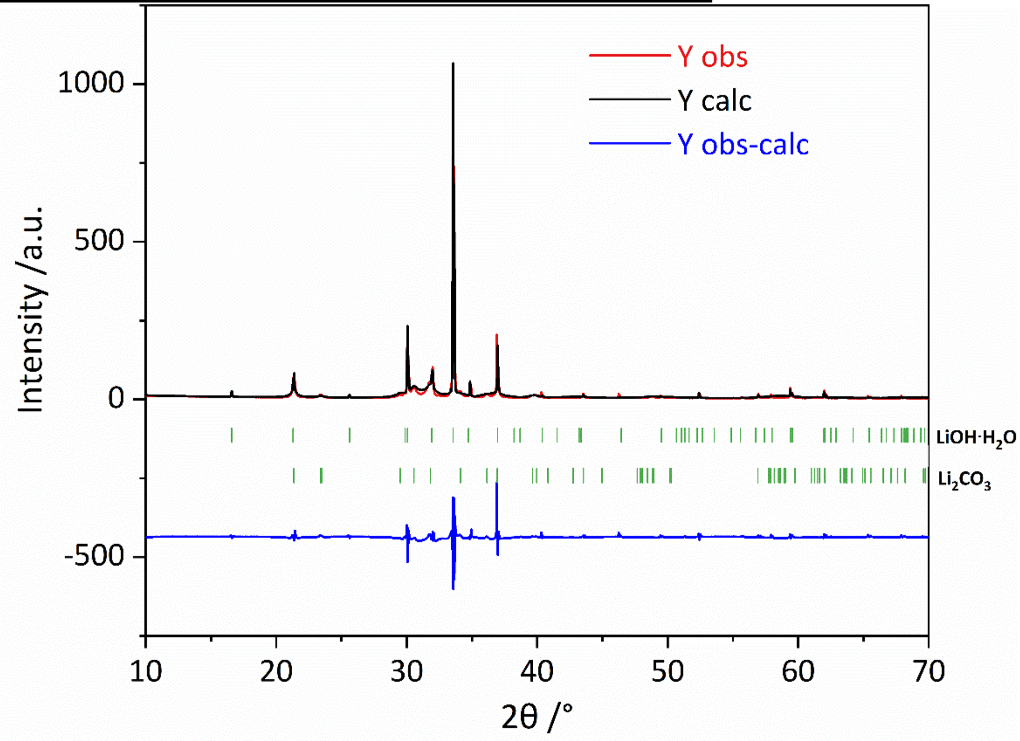

Fig. S2 Refinement of $LiOH{\cdot}H_2O$ as used in the synthesis of the layered oxides.

The $LiOH{\cdot}H_2O$ (Fischer Chemical, purity: ≥ 99%) that we used shows some carbonate impurities. The fraction is between 2% and 4% (see refinement below), but the error of the phase fraction of +/-2% is quite high.

Determining the exact value of the Li_2CO_3 content in pristine $LiOH{\cdot}H_2O$ is difficult because carbonate reflections are relatively low and broad. Moreover, they become narrower (and thus better visible) when the temperature increases upon synthesis, see Fig. S3. To address this, we first refined Li_2CO_3 with Le Bail and later as a normal phase upon the operando synthesis. This narrowing of the reflections indicates that Li_2CO_3 exists as a more amorphous impurity on the surface at the beginning. With the heat treatment, water is released from LiOH, and Li_2CO_3 crystallizes or goes through lattice perfection (see Fig. S3).

The presence of carbonate impurities upon the synthesis of layered oxide is reported, already [1,2]. The purity of the salt is graded > 99%, but it explicitly says the values are based on metal impurities. It also makes no difference whether or not the salt is opened in the glove box with an air-free transfer to the beamline. Nevertheless, the Li surplus in the synthesis is in the range of the carbonate impurities. At 800 °C, the carbonate vanishes. At this point, Li/Me disorder is still ~ 8%, which could also be due to an incomplete Li intercalation.

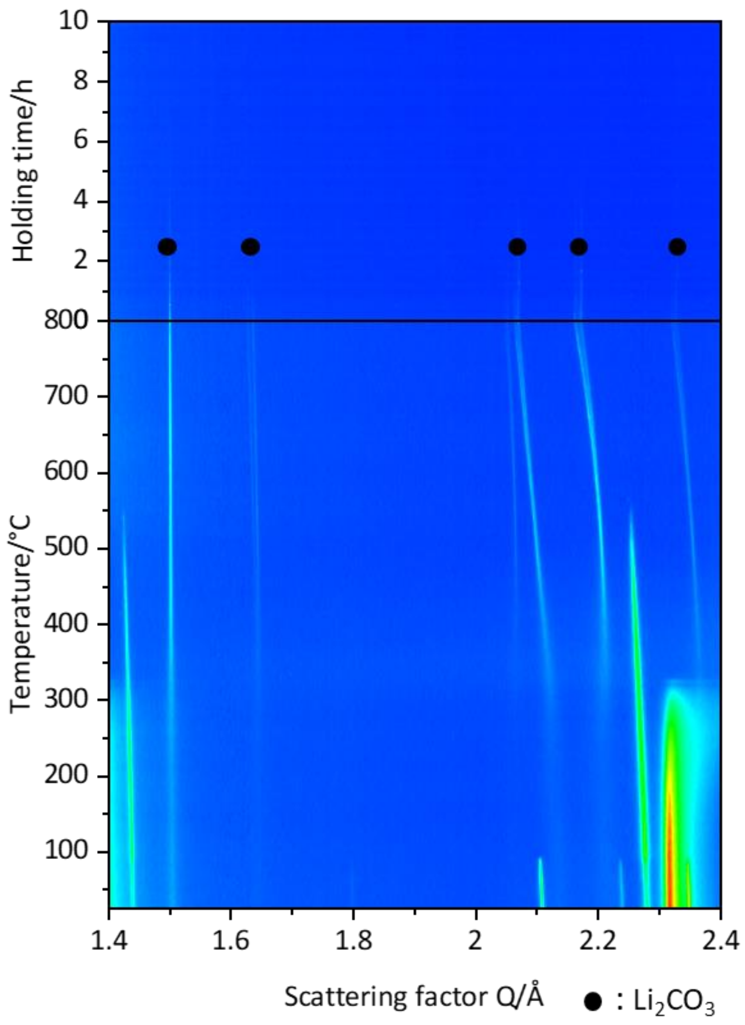

Fig. S3 Changes of the carbonate reflections upon NCM811 synthesis.

Choice of the holding steps

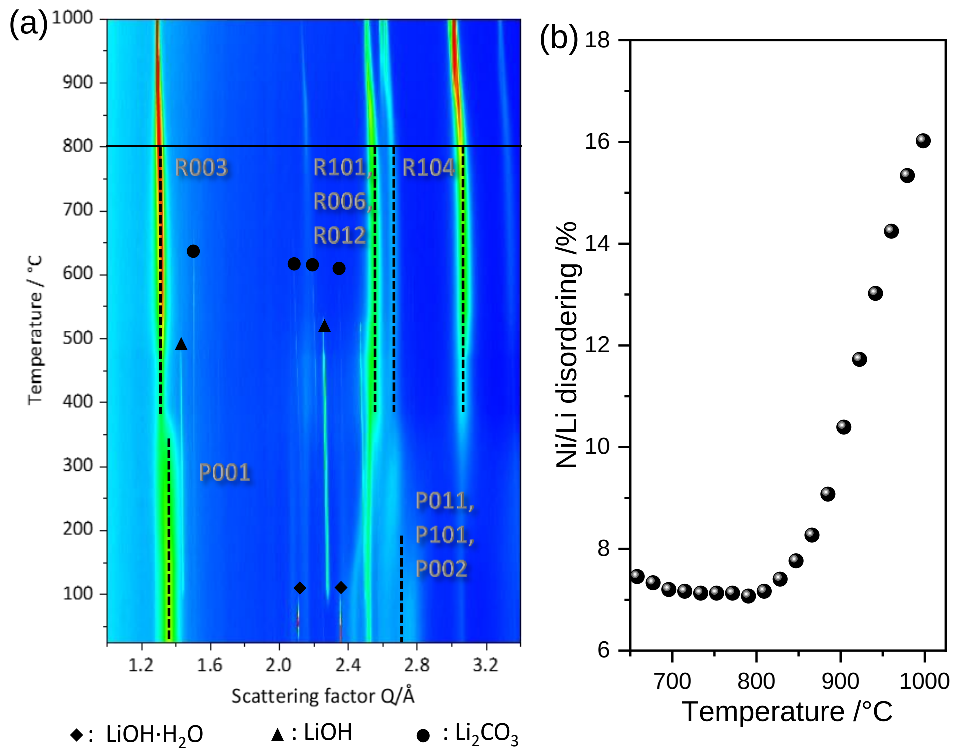

Fig. S4 In situ SXPD data of NCM111, collected upon calcination heating from RT to 1000 °C with a heating rate of 4 °C/min; B) The Ni/Li disorder vs. temperature from 650 °C to 1000 °C.

The optimum temperature for the second holding step was investigated with *operando* powder diffraction for NCM111 (Fig. S4.). A 1:1.03 mixture of the NCM111-precursor and LiOH was heated to 1000 °C while the evolution of Li/Me disorder was monitored. Until 800 °C, the Li/Me disorder decreases, but above 800 °C, the Li/Me disorder increases again, although the aim is to minimize it. The optimum in the final calcination temperature is also confirmed in literature [3,4].

Prior to this work, the 1st holding step was systematically investigated in [3] (Fig. S4). Applying a holding step below the melting point of LiOH leads to relatively high a, b- and c-lattice parameters. While the a, b-lattice parameter is striving towards a minimum due to Ni oxidation from ionic Ni^{2+} to covalent Ni^{3+} [5,6], the c-lattice parameter reaches a minimum due to the reduction of Li/Me disorder. A holding step above 550 °C leads to an imperfection in the lattice of Ni-rich layered oxides manifested in a higher a, b-lattice parameter of the structure. XPS measurements show that the oxidation of Ni remains incomplete, which led to the conclusion that Ni oxidation is a critical process around 500 °C. It does not proceed entirely if the temperature is raised too fast. The origin might be, that Li is too volatile at higher temperatures to enforce oxidation.

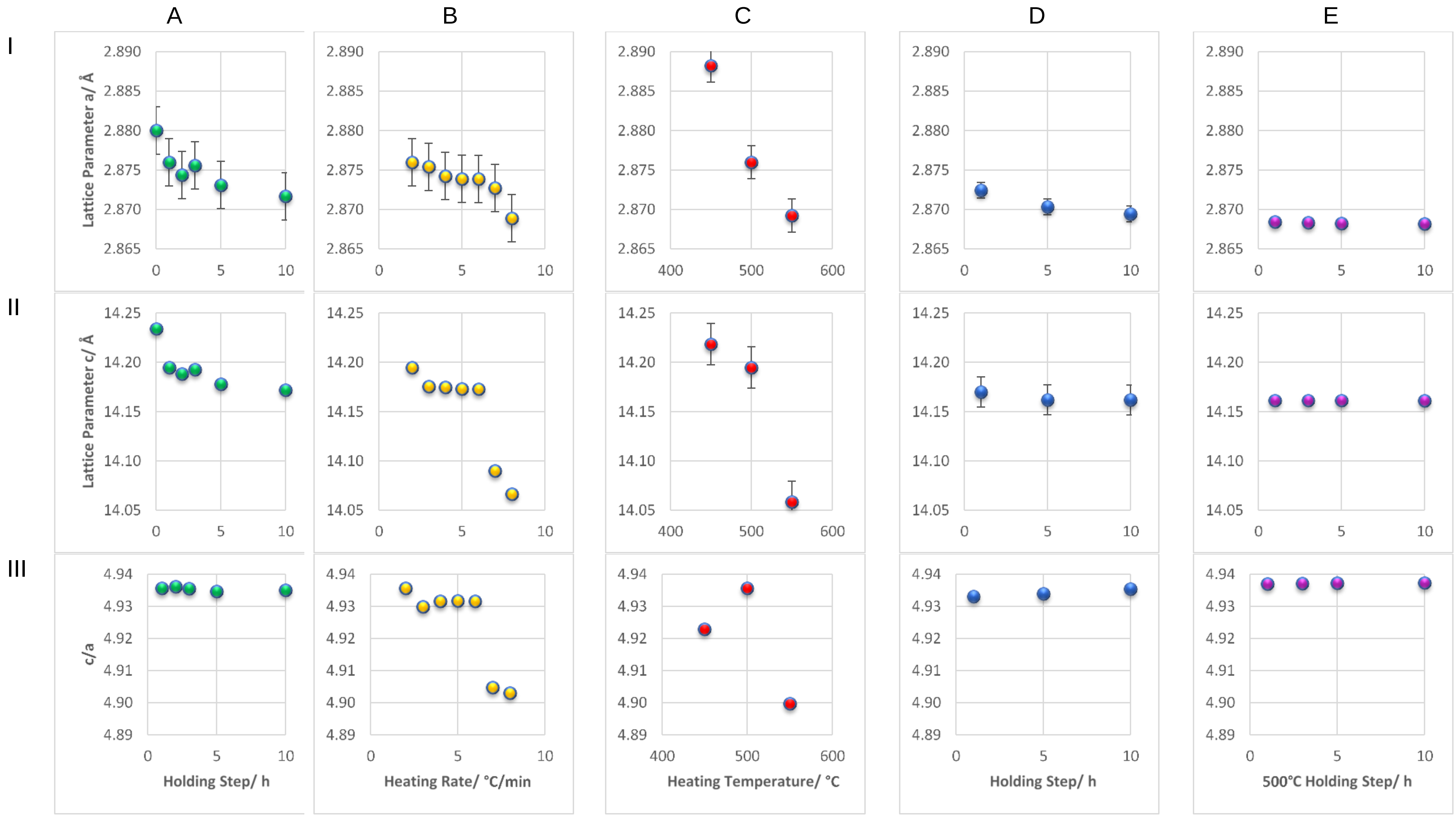

Fig. S5 Lattice parameter a (=b) (I), lattice parameter c (II), and the ratio c/a of NCM811. (A) was heated with one holding step at 500°C for different durations. (B) was heated with one holding step at 500°C with different heating rates. (C) was heated with one holding step at 450°C, 500°C, and 550°C. (D) was heated with one holding step at 800°C for different durations. (E) was heated with two holding steps; the 800°C holding step was fixed to 10 h, and the 500°C holding step was varied.

In situ SXPD setup at I11

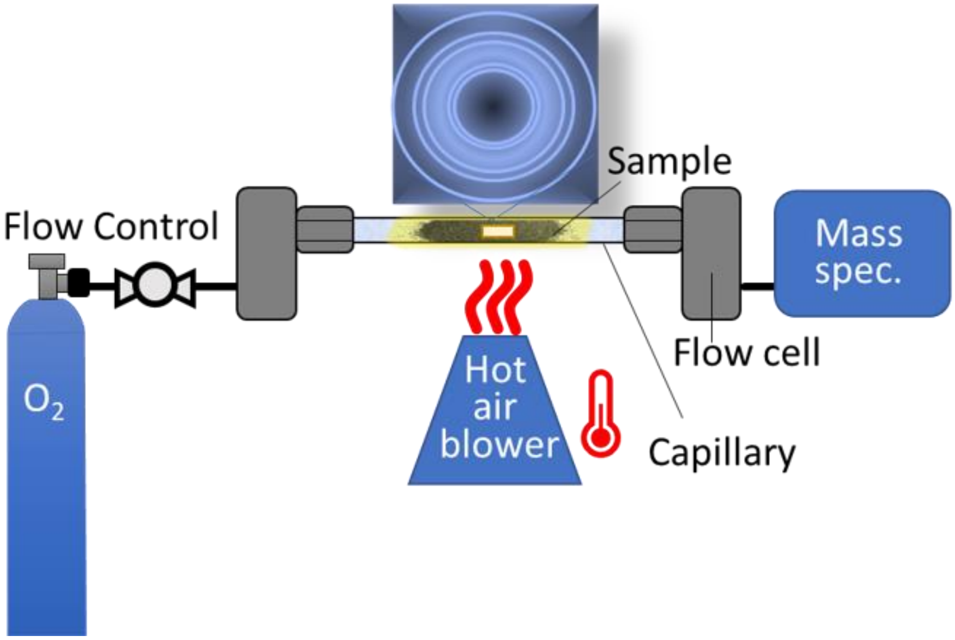

Fig. S6 Schematic sketch of the in situ SXPD setup at beamline I11. The beam is in the viewing direction.

A sapphire capillary (diameter: 0.5 mm) was used to withstand the high temperature, which can contribute to diffraction peaks in the data. The capillary was loaded with the synthesized precursor, premixed with $LiOH \cdot H_2O$ in a 1:1.03 molar ratio. For the synthesis of NCM811, O_2 was flushed through the capillary with the outlet connected to a mass spectrometer to determine the concentration of H_2O and O_2 released upon the synthesis. For the synthesis of NCM111, the sample capillary was left open to the air. As the process was performed in air, the water content in the atmosphere dominates the H_2O release from the precursor-lithium hydroxide calcination. It was, therefore, not useful to obtain the corresponding MS data. A Cyberstar Hot Air Blower was used to heat up the capillary sample from room temperature to 800 °C at a rate of 4 °C/min. Diffraction patterns were measured throughout the heating and holding steps using a position-sensitive detector (PSD) [7]. With a wide-angle aperture (90°) and fast data collection of the detector, a whole diffraction pattern is measured in 90 s.

Data analysis of SXPD patterns

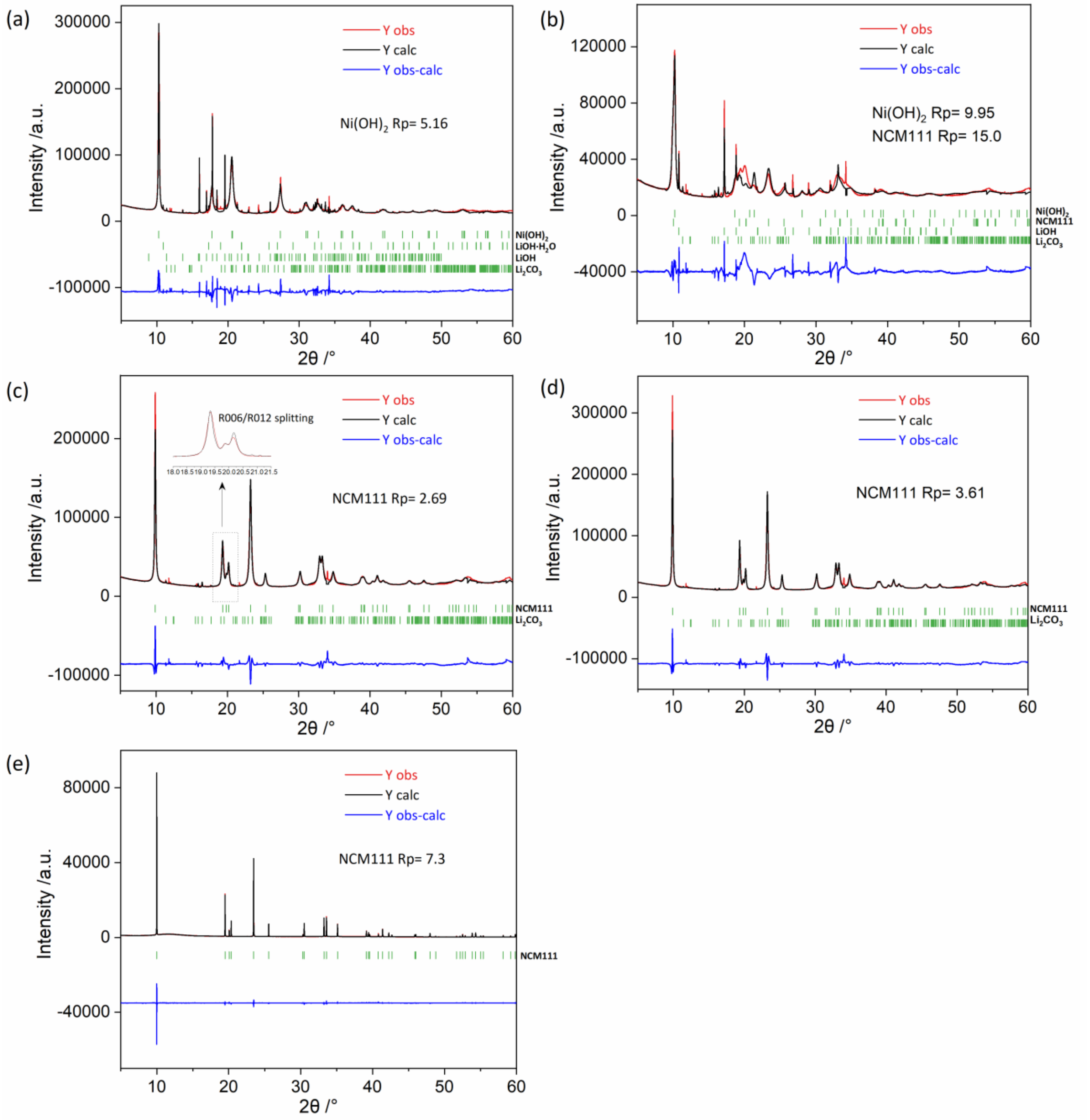

Fig. S7 Rietveld refinement analyses of SXPD data of Hold-NCM111, collected upon calcination, λ = 0.826556(2) Å. a) at 25°C; b) at 340°C; c) at 800°C; d) after annealing but without furnace cooling, e) after furnace cooling. (LXPD, λ =1.5406 Å).

In the plots, red lines are used for the observed data, black lines for calculated data, green bars for Bragg positions, and blue lines for the difference between the experimental and calculated data.

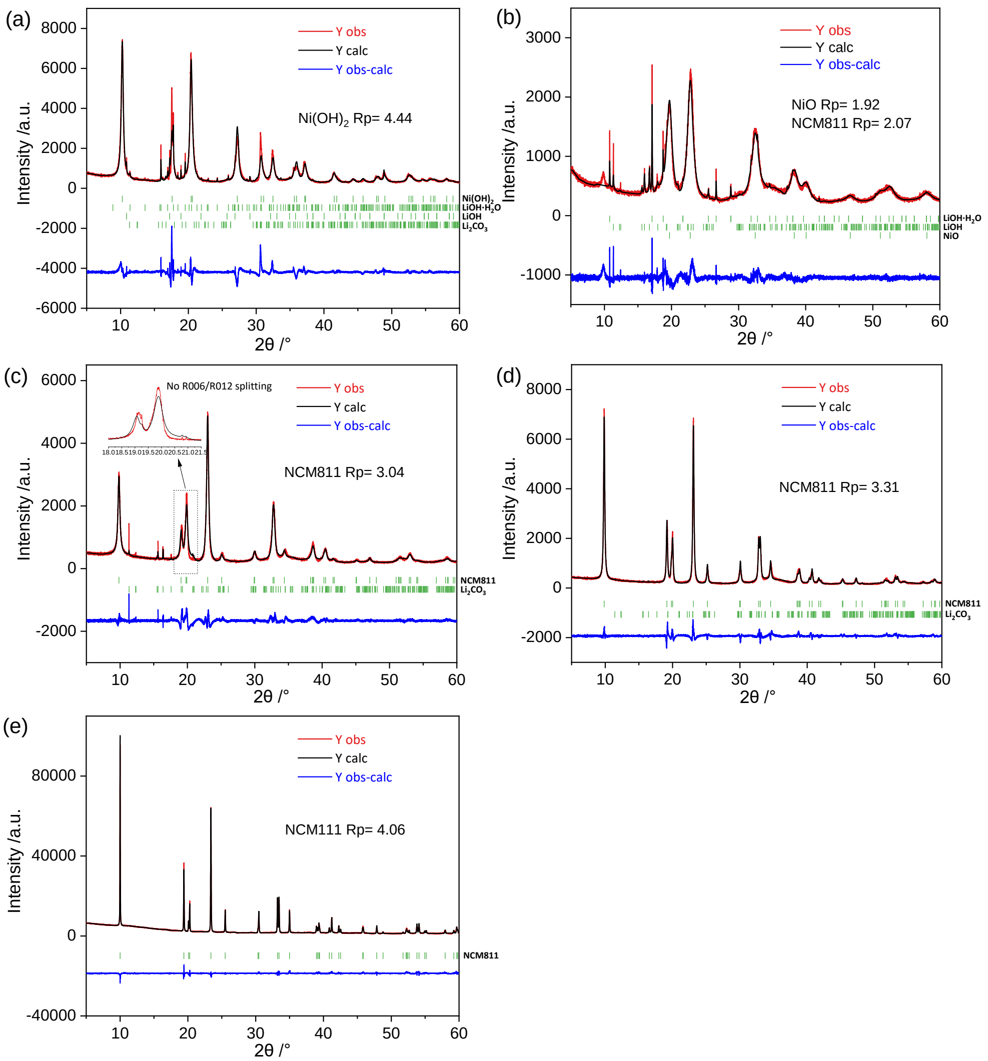

Fig. S8 *In situ* SXPD data of NCM 811, collected upon calcination, λ =0.826562(2) Å. a) at 25°C; b) at 400°C; c) at 800°C; d) after annealing but without furnace cooling, e) after furnace cooling. In the plots, red lines are used for the observed data, black lines for calculated data, green bars for Bragg positions, and blue lines for the difference between the observed and calculated data.

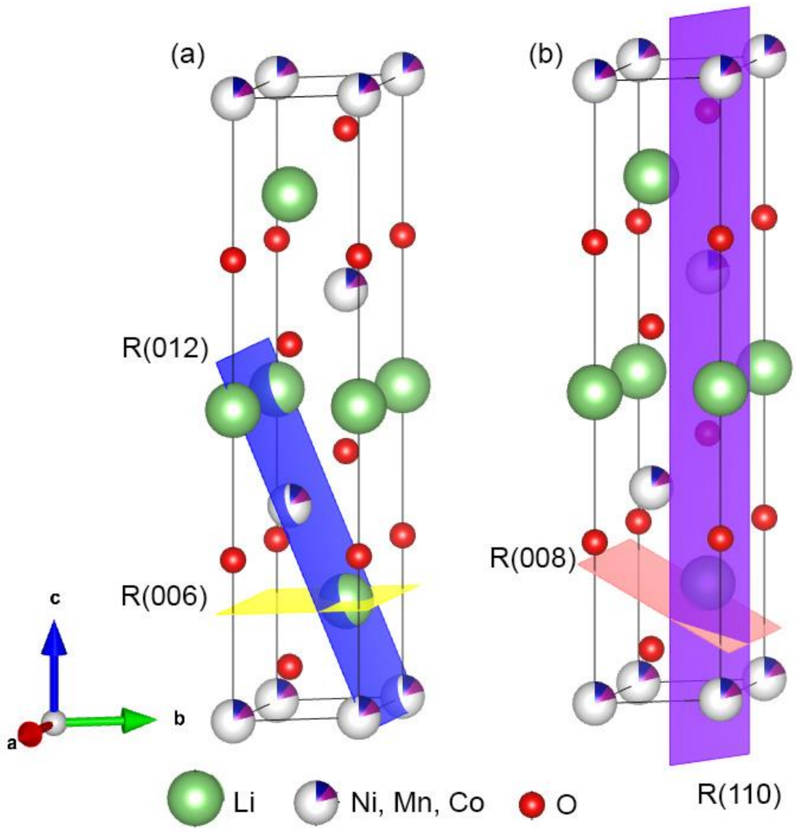

Fig. S9 a) Structures of NCM with the a) R(006), R(012) planes, and b) R(018), R(110) planes. The structure is produced by the software VESTA[8], and crystallographic data files [9].

Primarily, upon the synthesis of NCM811, in more detail upon the phase transformation from the cubic towards the rhombohedral structure, new reflections appear (or reflections split up). With Li intercalation, the symmetry of the cubic structure with a=b=c is broken. New reflections appear (R006, R012, R018, R110) because the atomic distances in the c-direction change; thus, the d-spacing between lattice spaces does. This is, e.g., the case for R012/R006, where R006 represents the d-spacing in the c-direction between Li and Me layers while R012 is determined by a and b only.

SEM and TGA data of NCM111-OH and NCM811-OH

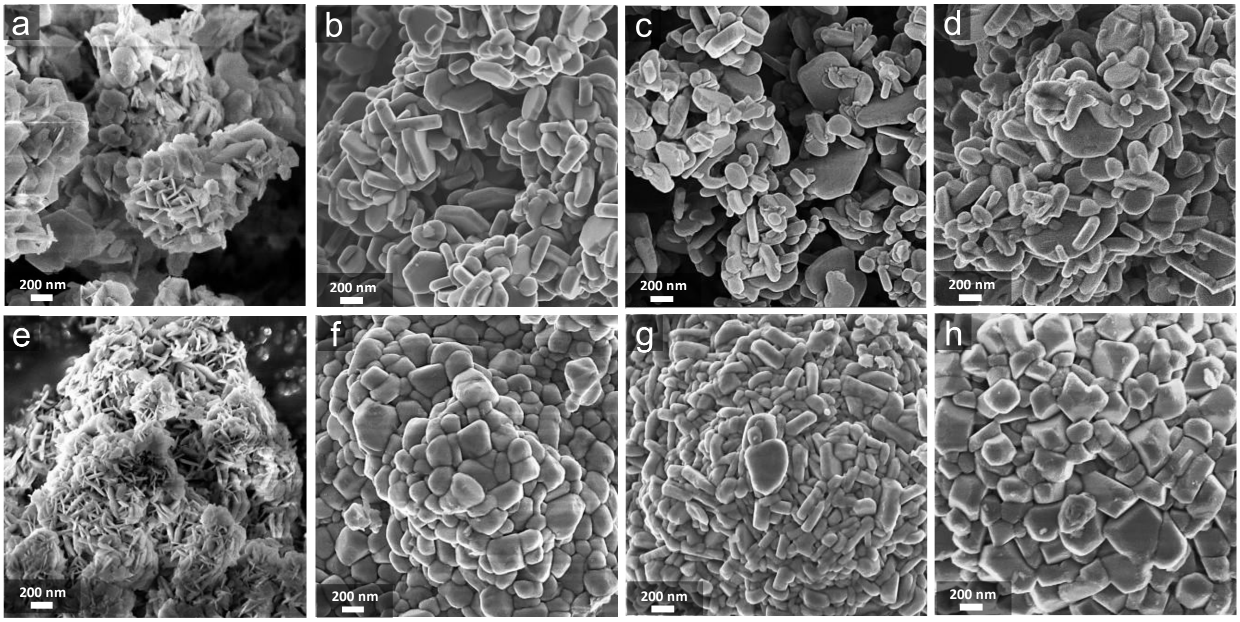

Fig. S10 SEM images of a) NCM111-OH; b) Hold-NCM111; c) Pre-NCM111; d) Comb-NCM111; e) NCM811-OH; f) Hold-NCM811; g) Pre-NCM811; h) Comb-NCM811.

The flakes of the precursors agglomerate together, forming flower-like particles. After the calcination, these precursors transform into the typical hierarchical secondary textured particles of micrometer-sizes, which embody densely packed primary particles of 100-200 nm. Hold-NCM811 and Comb-NCM811 have granular secondary particles, while Pre-NCM811 is made of plate-like secondary particles, and the morphology is poorly developed with large size distribution.

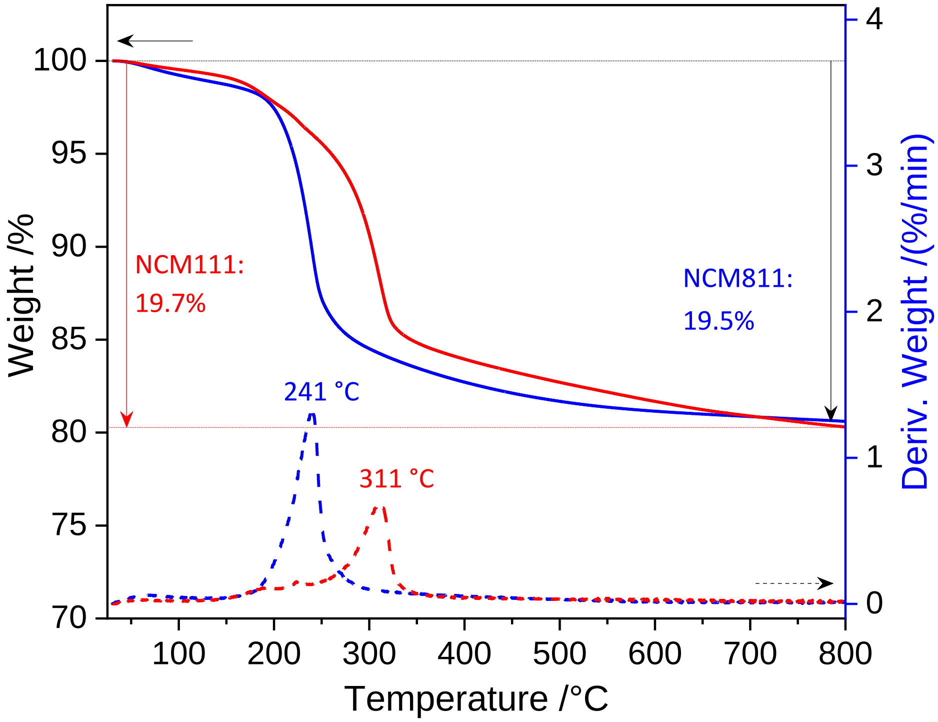

Fig. S11 The thermal stabilities of the precursors were investigated by thermogravimetric analysis (Thermogravimetric Analyzer Q5000 IR) for which the precursors were heated to 800 °C with a heating rate of 4 °C/min in air.

Additional Electrochemical data of NCM111 and NCM811

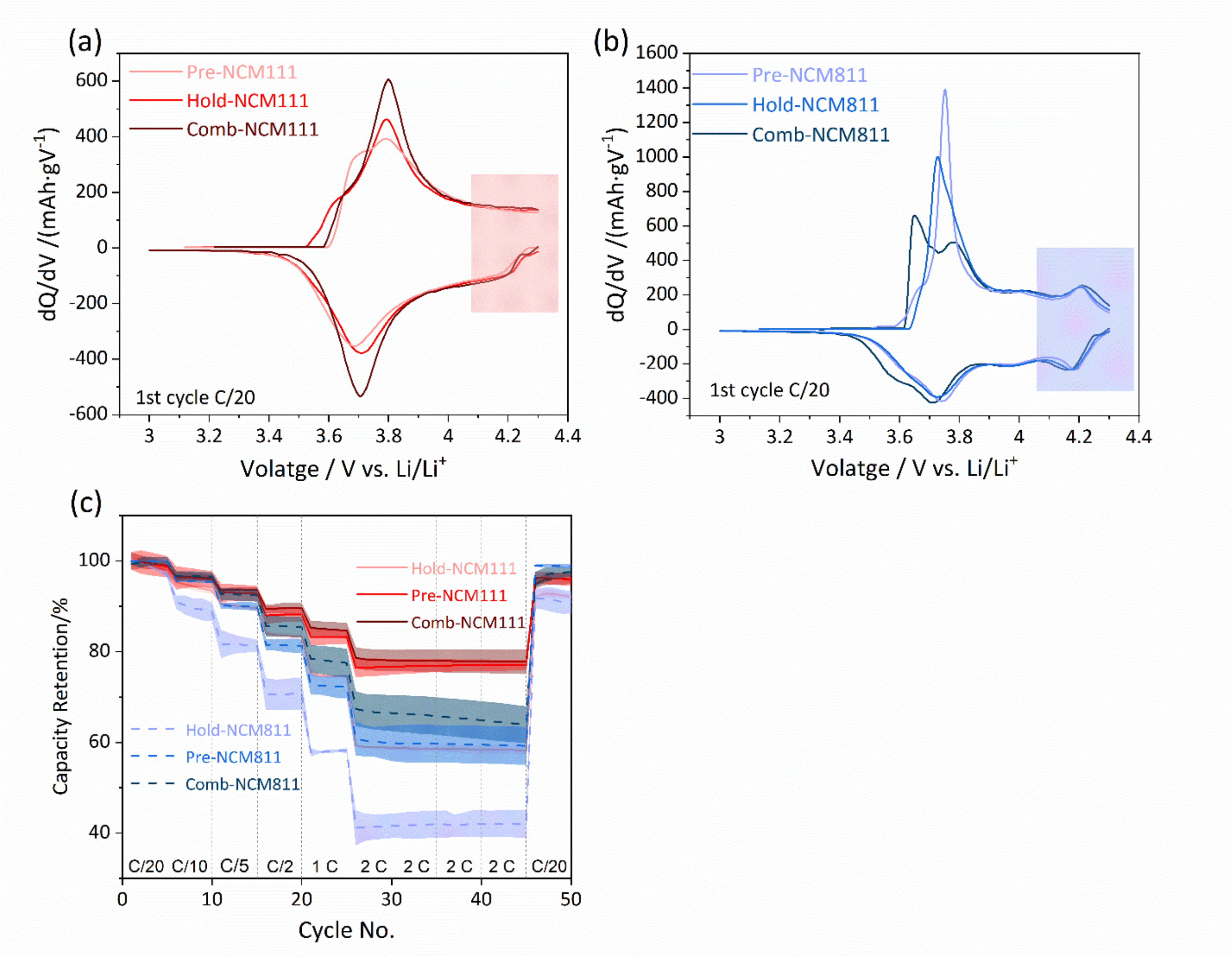

Fig. S12 dQ/dV curve of a) different NCM111s at 1st cycle, b) different NCM811s at 1st cycle, c) Capacity retention as a function of the C-rate for NCM811s and NCM111s.

Mass spectrosmetry data of NCM111 obtained upon calcination

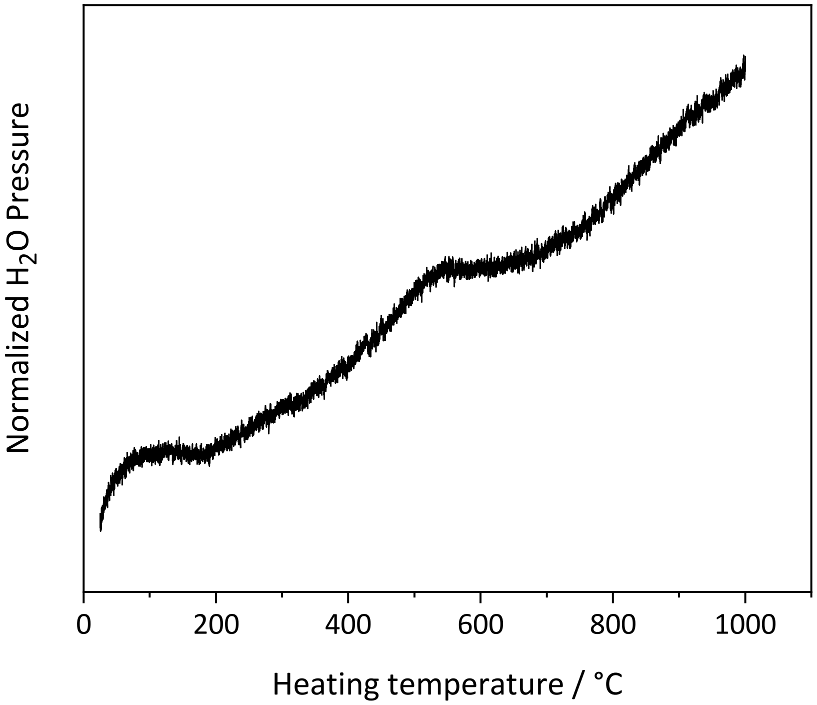

Fig. S13 Mass spectroscopic data show the H_2O pressure vs. temperature obtained during the synthesis of NCM111. Since the capillary was left open to the air, the H_2O signal from the NCM111OH material is obscured because of the existence of H_2O in the air.

Depiction of reflection planes

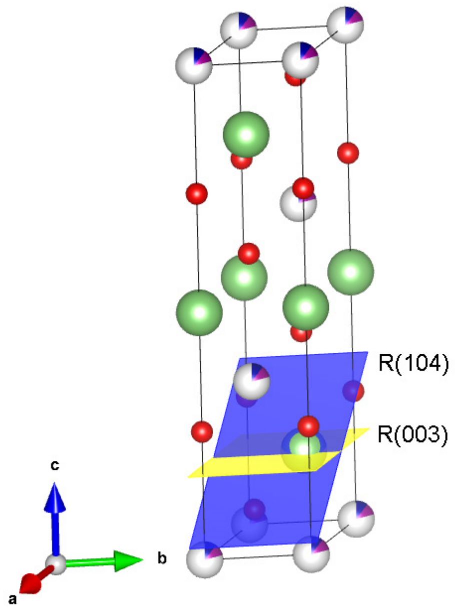

Fig. S14 Structures of NCM with the R(003) and R(104) planes. The structure is produced by the software VESTA [8] and crystallographic data files [9].

The relative intensity of the R003 and the R104 upon stage III reveals more detailed information about the lithium incorporation and cation ordering since the R003 represents the occupation of the transition metal layer (the mean electron density of the elements occupying the 3b sites), while the electron density of 3a and 3b sites produced R104 reflection.

Details about Rietveld refinement

The multiple-phase refinements were performed scan-by-scan. The main phases (phases with the highest content, see Fig. 6) were always refined first and the other phases were added one by one according to their weight. The Thompson-Cox-Hastings pseudo-Voigt convoluted with axial divergence asymmetry function was used as the normalized peak shape function. The instrumental reflection broadening parameters (U, V, W) were determined separately by using a Si NIST standard as described in the experimental section. These parameters were included in the refinement using an instrumental resolution file (.irf) file. Upon the refinement the parameters are refined according to the following sequence:

1. The background cannot be described with a polynomial function due to contributions from the capillary. Instead, background interpolation and subtraction were used for the refinements. Therefore we have chosen 30 points on the background which represent the progression as described in detail in [6].
2. The zero shift was refined in the initial scans and was fixed for the subsequent scans since the position of the capillary did not change upon calcination.
3. The scale factors of all phases excluding $LiOH{\cdot}H_2O$ at temperatures < 120°C were refined and were left to be free.
4. The lattice parameters a, b, and c of all the phases were refined according to their space group. If the phase content decreased below 5% sometimes the values needed to be fixed. Special refinement of lattice parameters was also necessary in the case of distinguishing NiO and $R\overline{3}m$ reflections. Details can be found in Fig. S15 and Fig. S16.
5. The overall Debye-Waller factor (Bov) was refined.
6. The sample parameters (X, Y) of all phases were refined.
7. The site occupancies of Li1, Ni1, Li2, and Ni2 of the $R\overline{3}m$ phase were refined simultaneously. Thereby the values were constrained since Li ions and Ni ions just exchanged positions but the overall amount was fixed according to the remaining LiOH content.

The Le Bail method was used for $LiOH{\cdot}H_2O$ at temperatures < 120 °C because of its preferred orientation. The rock salt-type structure ($Fm\overline{3}m$) was introduced over 500 °C and was omitted in the refinements at temperatures > 700 °C as discussed in the main text. At 500 °C, the refinement significantly improves by adding two transition metal oxide phases ($Fm\overline{3}m$ and $R\overline{3}m$), while at 800 °C, one phase refinement ($R\overline{3}m$) is more suitable, see Fig. S15. Initially, reflections that are transferred from the NiO phase into the $R\overline{3}m$ phase appear broader than reflections introduced solely due to the separation of Ni and Li into different layers. The rock salt-type structure was omitted in the refinements at temperatures > 700 °C because of reflection broadening of reflections with both contributions ($R\overline{3}m$ and $Fm\overline{3}m$) and only the $R\overline{3}m$ contribution followed a single Williamson Hall line (See Fig. S15 and S16).·If a Williamson Hall plot of the reflection which is only present in the $R\overline{3}m$ phase is made, the reflection broadening of the other reflections ($R\overline{3}m$ and $Fm\overline{3}m$) do not lie on this line. Instead, they form a new line with a different slope, see Fig. S16.

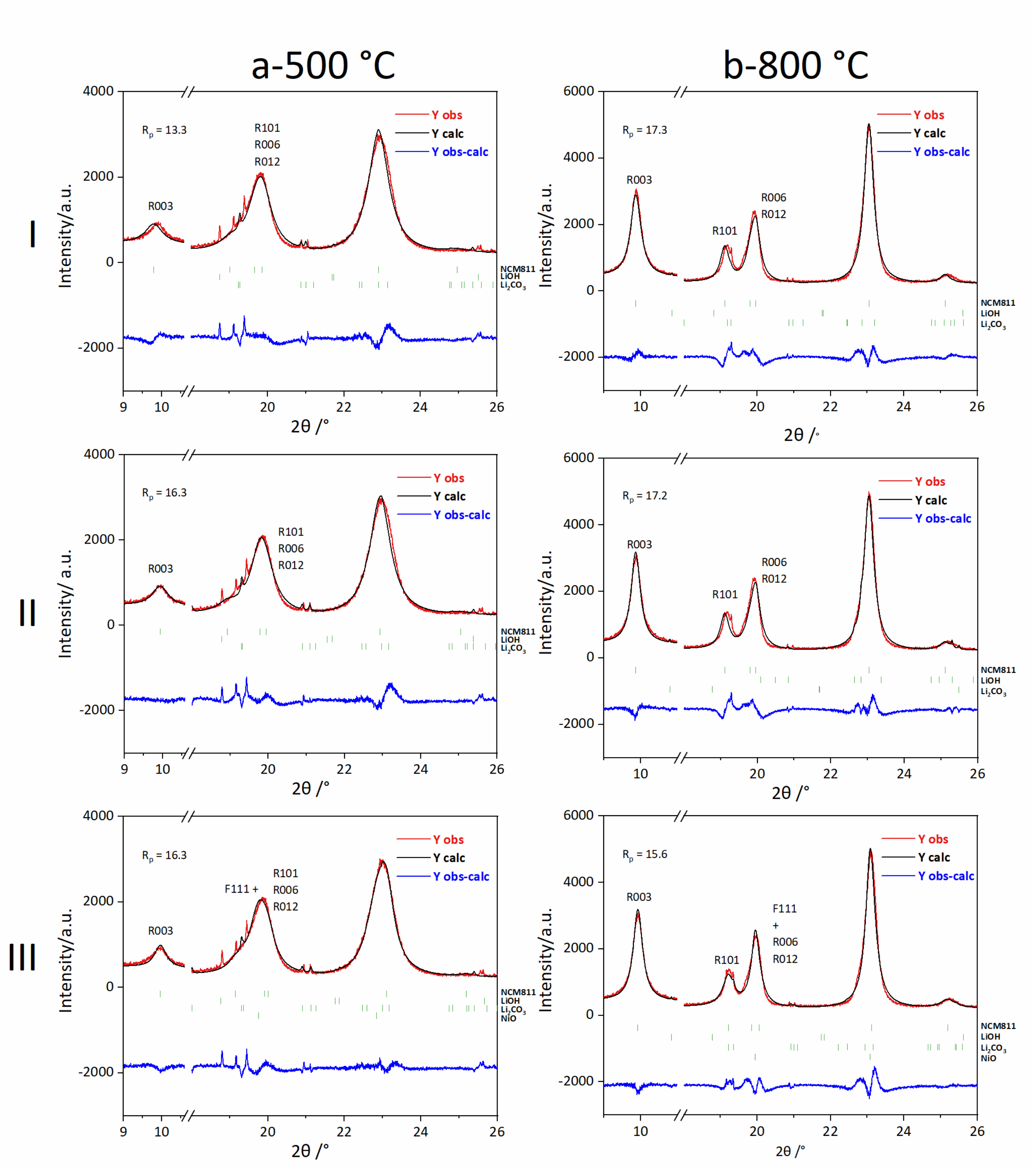

Fig. S15 Rietveld refinement analysis of SXPD patterns for Hold-NCM811 at 500°C: Ia) refining the pattern with one phases (NCM811), IIa) using one phase (NCM811) with anisotropy, IIIa) using two phases (NiO and NCM811). Rietveld refinement analysis of SXPD patterns for Hold-NCM811 at 800°C: Ib) refining the pattern with one phases (NCM811), IIb) using one phase (NCM811) with anisotropy, IIIb) using two phases (NiO and NCM811).

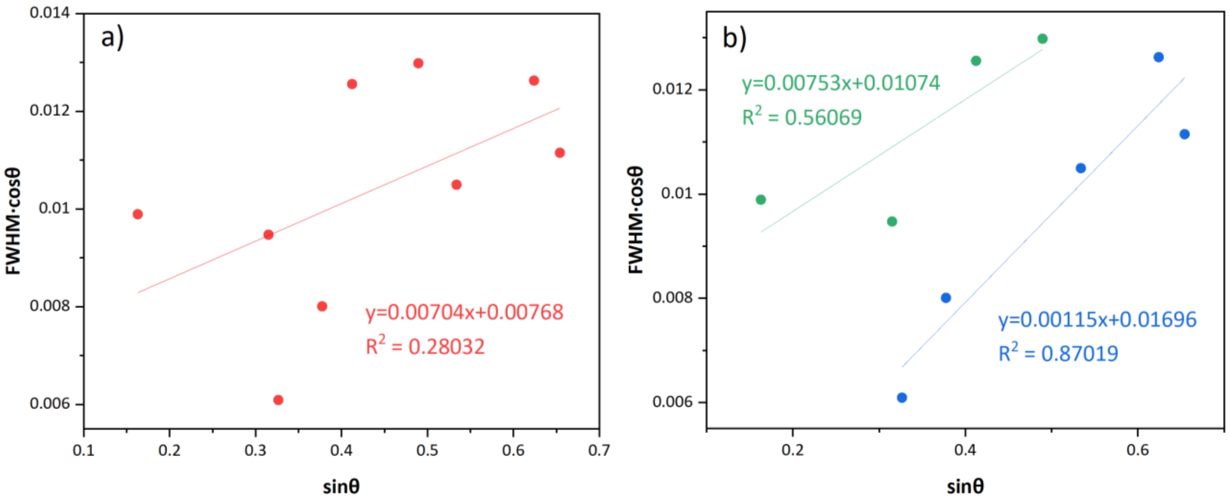

Fig. S16 Williamson Hall plot from the powder diffration data of NCM811 and LiOH·H_2O heated to 500 °C. a) shows the plot using one regression line while b) is separated into reflections that are only present in the rhombohedral phase and reflections that are present in both phases.

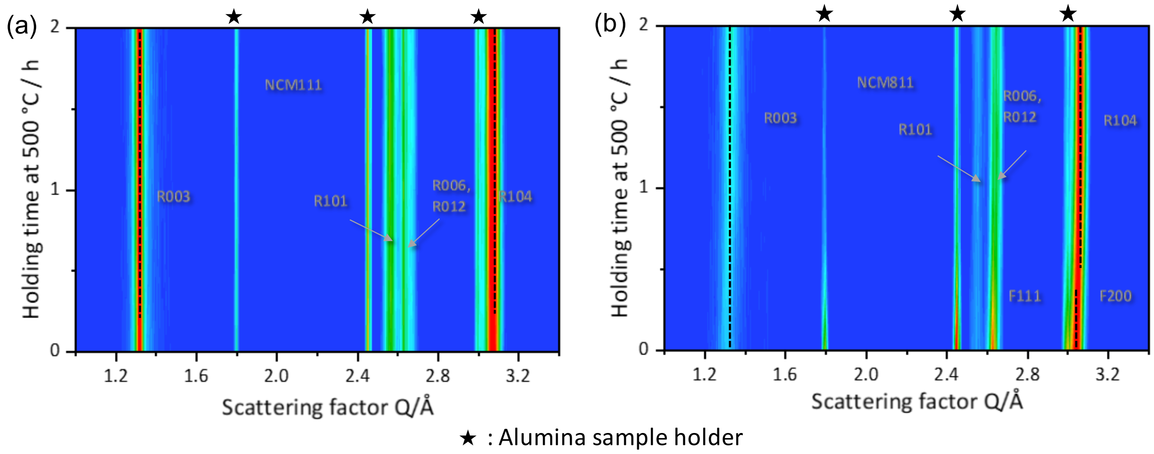

Fig. S17 Contour plots of XRD patterns upon pre-annealing of a) Pre-NCM111; b) Pre-NCM811, λ =0.727140(1) Å.

In order to give the reader a better understanding of how the refinement of the data was performed, the following tables give parameters used and obtained upon data processing. Rp values are given for the most important refinements. They reflect the difference between the observed and calculated structure factors normalized to the observed values, multiplied by their multiplicity. Note that Rp values are not actively included to minimize mismatches in the refinement.

Tab. S1 Crystallographic parameters of all phases [13-18] used in this work.

Name	site	x	y	z	Fract.
$Ni(OH)_2$ Cell parameters					
Space group: *P-3m1*, *a* = *b* = 3.1268 Å, *c* = 4.6060 Å, gamma = 120°, V = 39 Å^3, *Z* = 1					
Atomic positions					
Name	site	x	y	z	Fract.
Ni1	1a	0.0000	0.0000	0.0000	1.0000
O1	2d	0.3333	0.6667	0.2153	1.0000
H1	2d	0.3333	0.6667	0.4537	1.0000
NiO Cell parameters					
Space group: *Fm-3m*, *a* = *b* = *c* = 4.178 Å, V = 72.93 Å^3, *Z* = 4					
Atomic positions					
Name	site	x	y	z	Fract.
Ni1	4a	0.0000	0.0000	0.0000	1.0000
O1	4b	0.5000	0.5000	0.5000	1.0000
$LiNi_{0.8}Co_{0.1}Mn_{0.1}O_2$ Cell parameters					
Space group: *R-3m*, *a* = *b* = 2.8645 Å, *c* = 14.161 Å, gamma = 120°, V = 100.63 Å^3, *Z* = 3					
Atomic positions					
Name	site	x	y	z	Fract.
Li1	3b	0.0000	0.0000	0.5000	0.9800
Ni1	3b	0.5000	0.5000	0.5000	0.0200
Li2	3a	0.0000	0.0000	0.0000	0.0500
Ni2	3a	0.5000	0.5000	0.5000	0.75000
Co1	3a	0.0000	0.0000	0.0000	0.1000
Mn1	3a	0.0000	0.0000	0.0000	0.1000
O1	6c	0.5000	0.5000	0.2590	1.0000
$LiNi_{0.33}Co_{0.33}Mn_{0.33}O_2$ Cell parameters					
Space group: *R-3m*, *a* = *b* = 2.86 Å, *c* = 14.227 Å, gamma = 120°, V = 100.78 Å^3, *Z* = 3					
Atomic positions					
Name	site	x	y	z	Fract.
Li1	3b	0.0000	0.0000	0.5000	0.9750
Ni1	3b	0.5000	0.5000	0.5000	0.0250
Li2	3a	0.0000	0.0000	0.0000	0.025
Ni2	3a	0.5000	0.5000	0.5000	0.3090
Co1	3a	0.0000	0.0000	0.0000	0.3333
Mn1	3a	0.0000	0.0000	0.0000	0.3333
O1	6c	0.5000	0.5000	0.2411	1.0000
$LiOH{\cdot}H_2O$ Cell parameters					

Space group: *C12/m1*, *a* = 7.37 Å, *b* = 8.26 Å, *c* = 3.19 Å, beta = 110.3°, V = 182.13 Å^3, *Z* = 4

Atomic positions

Name	site	x	y	z	Fract.
Li1	4h	0.0000	0.3474	0.3952	1.0000
O1	4i	0.2857	0.0000	0.3952	1.0000
O2	4h	0.0000	0.2066	0.0000	1.0000
H1	4i	0.2370	0.0000	0.6310	1.0000
H2	8j	0.1070	0.1180	0.0040	1.0000

LiOH· Cell parameters

Space group: *P4/nmm*, *a* = *b* = 3.549 Å, *c* = 4.334 Å, V = 54.59 Å^3, *Z* = 2

Atomic positions

Name	site	x	y	z	Fract.
Li1	2a	0.0000	0.0000	0.0000	1.0000
O1	2c	0.0000	0.5000	0.1938	1.0000
H1	2c	0.0000	0.5000	0.4100	1.0000

Li_2CO_3· Cell parameters

Space group: *C12/c1*, *a* = 8.35884 Å, *b* = 4.97375 Å, *c* = 6.19377 Å, beta = 114.789°, V = 233.78 Å^3, *Z* = 4

Atomic positions

Name	site	x	y	z	Fract.
Li1	8f	0.1968	0.4454	0.8334	1.0000
C1	4e	0.0000	0.0665	0.2500	1.0000
O1	4e	0.0000	0.0321	0.2500	1.0000
O2	8f	0.1463	-0.0631	0.3133	1.0000

Tab. S2 The lattice parameters and R_p values obtained upon data processing for the most important refinements of NCM111

NCM111	Space group (the main phase)	Lattice parameter a = b / Å	Lattice parameter c / Å	R_p
At 25°C;	$P\bar{3}m1$ (NCM111-OH)	3.09706(12)	4.61124(10)	5.16
At 340°C	$P\bar{3}m1$ (NCM111-OH)	2.92935(35)	4.57527(33)	9.95
	$R\bar{3}m$ (NCM111)	2.88267(35)	14.06023(177)	15.0
At 800°C	$R\bar{3}m$ (NCM111)	2.88213(5)	14.36425(51)	2.69
After 800 °C (without cooling)	$R\bar{3}m$ (NCM111)	2.87996(4)	14.37670(46)	3.61
Final product	$\boldsymbol{R\bar{3}m}$ (NCM111)	2.86009(0)	14.22683(2)	7.3

Tab. S3 Disorder and the lattice parameters of the NCM811s obtained with different cooling procedures. Quenching: sample cooling with liquid nitrogen quenching, Furnace: sample cooling in the tube furnace.

Sample	Holding at 800 °C	Cooling	Disorder value / %	Lattice parameter a=b / Å	Lattice parameter c / Å
Without 500 °C pre-annealing step	0h	Quenching	8.48	2.873072	14.192163
	0h	Furnace	7.75	2.872836	14.19306
	10h	Quenching	5.01	2.872119	14.203515
	10h	Furnace	4.80	2.872092	14.206037

Tab. S4 The mean crystalline size and microstrain factor Y of NCMs upon calcination. The mean crystalline sizes are calculated according to the Scherrer equation $L = \frac{K\lambda}{\beta \cos\theta}$ [10]; L: the mean size of the crystalline size; K: Scherrer constant; λ: the X-ray wavelength; β: the line broadening at half the maximum; θ: the Bragg angle. The microstrain factor Y is the Lorentzian strain broadening contribution in the Equation $H_L = X \tan\theta + Y/\cos\theta + Z$ [11, 12].

Materials	Temperature	The mean crystalline size/nm	Microstrain factor Y
	500°C	5.53	0.254
NCM111	800°C	11.14	0.120
	After cooling	13.91	0.025
	500°C	1.81	0.379
NCM811	800°C	2.56	0.323
	After cooling	12.1	0.012

Tab. S 1 The lattice parameters and R_p values obtained upon data processing for the most important refinements of NCM811

NCM811	Space group (the main phase)	Lattice parameter a=b / Å	Lattice parameter c / Å	R_p
At 25°C;	$P\bar{3}m1$ (NCM811-OH)	3.12330(6)	4.63008(13)	4.44
At 400°C	$Fm\bar{3}m$(Rock salt-type phase)	4.04585(12)	/	1.92
	$R\bar{3}m$ (NCM811)	2.91400(47)	14.40978(355)	2.07
At 800°C	$R\bar{3}m$ (NCM811)	2.91680(4)	14.39708(39)	3.04
After 800 °C (without cooling)	$R\bar{3}m$ (NCM811)	2.90263(2)	14.36921(16)	3.31
Final product	$\boldsymbol{R\bar{3}m}$ (NCM811)	2.87171(1)	14.20421(4)	4.06

Tab. S5 The disorder values and the lattice parameters of the NCM811s were obtained with different cooling procedures. Quenching: sample cooling with liquid nitrogen quenching, Furnace: sample cooling in the tube furnace.

Sample					
	Holding at 800 °C	Cooling	Disorder value / %	Lattice parameter a=b / Å	Lattice parameter c / Å
Without 500 °C pre-annealing step	0h	Quenching	8.48	2.873072	14.192163
	0h	Furnace	7.75	2.872836	14.19306
	10h	Quenching	5.01	2.872119	14.203515
	10h	Furnace	4.80	2.872092	14.206037

NEXAFS data for hold-NCM811

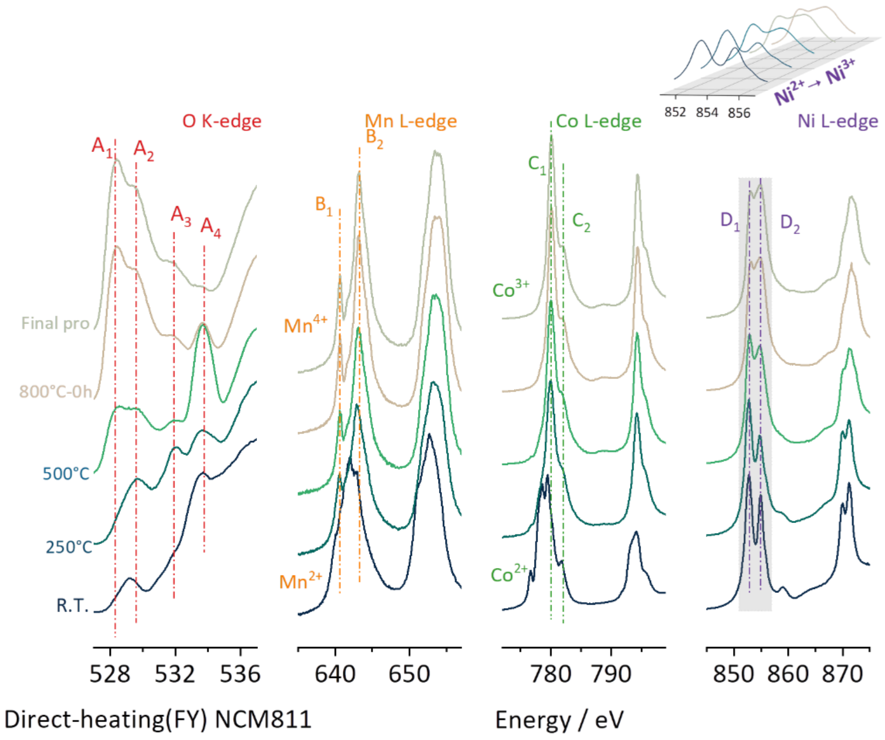

Fig. S18 soft NEXAFS spectra of O K edge, Mn L edge, Co L edge, and Ni L edge for NCM811s at different temperatures. The NCM811s are obtained with the heating program Fig. 1a.

Reference

(1) Duan, Y.; Yang, L.; Zhang, M.-J.; Chen, Z.; Bai, J.; Amine, K.; Pan, F.; Wang, F., Insights into Li/Ni ordering and surface reconstruction during synthesis of Ni-rich layered oxides. *J. Mater. Chem. A.* 2019, 7 (2), 513-519.

(2) Zhao, J.; Zhang, W.; Huq, A.; Misture, S. T.; Zhang, B.; Guo, S.; Wu, L.; Zhu, Y.; Chen, Z.; Amine, K., In Situ Probing and Synthetic Control of Cationic Ordering in Ni-Rich Layered Oxide Cathodes. *Adv. Energy Mater.* 2017, 7 (3), 1601266.

(3) Zheng, J.; Ye, Y.; Liu, T.; Xiao, Y.; Wang, C.; Wang, F.; Pan, F., Ni/Li disordering in layered transition metal oxide: electrochemical impact, origin, and control. *Accounts Chem. Res.* 2019, 52 (8), 2201-2209.

(4) Wang, D.; Kou, R.; Ren, Y.; Sun, C. J.; Zhao, H.; Zhang, M. J.; Li, Y.; Huq, A.; Ko, J. P.; Pan, F., Synthetic Control of Kinetic Reaction Pathway and Cationic Ordering in High-Ni Layered Oxide Cathodes. *Adv. Mater.* 2017, 29 (39), 1606715.

(5) Bröker, A.; Chemical and structural evolution upon the synthesis of layered oxides. *Master Thesis.* Münster University, Germany, 2021.

(6) Kleiner, K.; Murray, C. A.; Grosu, C.; Ying, B.; Winter, M.; Nagel, P.; Schuppler, S.; Merz, M., On the Origin of Reversible and Irreversible Reactions in LiNixCo (1− x)/2Mn (1− x)/2O2. *J. Electrochem. Soc.* 2021, 168 (12), 120533.

(7) Thompson, S. P.; Parker, J. E.; Marchal, J.; Potter, J.; Birt, A.; Yuan, F.; Fearn, R. D.; Lennie, A. R.; Street, S. R.; Tang, C. C., Fast X-ray powder diffraction on I11 at Diamond. *J. Synchrotron Radiat.* **2011,** *18* (4), 637-648.

(8) K. Momma and F. Izumi, "VESTA 3 for three-dimensional visualization of crystal, volumetric and morphology data," J. Appl. Crystallogr., **44**, 1272-1276 (2011).

(9) Li, L.-j.; Li, X.-h.; Wang, Z.-x.; Guo, H.-j.; Yue, P.; Chen, W.; Wu, L., Synthesis, structural and electrochemical properties of LiNi0. 79Co0. 1Mn0. 1Cr0. 01O2 via fast co-precipitation. J. Alloy. Compd. 2010, 507 (1), 172-177.

(10) Langford, J. I.; Wilson, A., Scherrer after sixty years: a survey and some new results in the determination of crystallite size. *Journal of applied crystallography* **1978,** *11* (2), 102-113.

(11) Kleiner, K.; Strehle, B.; Baker, A. R.; Day, S. J.; Tang, C. C.; Buchberger, I.; Chesneau, F.-F.; Gasteiger, H. A.; Piana, M., Origin of High Capacity and Poor Cycling Stability of Li-Rich Layered Oxides: A Long-Duration in Situ Synchrotron Powder Diffraction Study. *Chem. Mater.* **2018,** *30 (11),* 3656-3667.

(12) Fajar, A.; Gunawan, G.; Kartini, E.; Mugirahardjo, H.; Ihsan, M., Crystallite size and microstrain measurement of cathode material after mechanical milling using neutron diffraction technique. *Atom Indonesia* **2011,** 36 (3), 111-115.

(13) Alcock, N., Refinement of the crystal structure of lithium hydroxide monohydrate. *Acta Crystallogr. B: Struct. Crystallogr. Crystal Chem.* **1971,** 27 (8), 1682-1683.

(14) Mair, S., The electron distribution of the hydroxide ion in lithium hydroxide. *Acta Crystallogr. A: Cryst. Phys. Diffr. Theor. Gen. Crystallogr.* **1978,** 34 (4), 542-547.

(15) Idemoto, Y.; Richardson Jr, J. W.; Koura, N.; Kohara, S.; Loong, C.-K., Crystal structure of (LixK1−x) 2CO3 (x= 0, 0.43, 0.5, 0.62, 1) by neutron powder diffraction analysis. *J. Phys. Chem. Solids* **1998,** 59 (3), 363-376.

(16) Kazimirov, V. Y.; Smirnov, M.; Bourgeois, L.; Guerlou-Demourgues, L.; Servant, L.; Balagurov, A.; Natkaniec, I.; Khasanova, N.; Antipov, E., Atomic structure and lattice dynamics of Ni and Mg hydroxides. *Solid State Ionics*. **2010,** 181 (39-40), 1764-1770.

(17) SASAKI, S.; FUJINO, K.; TAKÉUCHI, Y., X-ray determination of electron-density distributions in oxides, MgO, MnO, CoO, and NiO, and atomic scattering factors of their constituent atoms. *Proc. Jpn. Acad. B* **1979,** *55* (2), 43-48.

(18) Yin, S.-C.; Rho, Y.-H.; Swainson, I.; Nazar, L., X-ray/Neutron Diffraction and Electrochemical Studies of Lithium De/Re-Intercalation in Li1-x Co1/3Ni1/3Mn1/3O2 (x= 0→ 1). *Chem. Mater.* **2006,** 18 (7), 1901-1910.
